# ﻿IMA GENOME - F20 A draft genome assembly of *Agroatheliarolfsii*, *Ceratobasidiumpapillatum*, *Pyrenopezizabrassicae*, *Neopestalotiopsismacadamiae*, *Sphaerellopsisfilum* and genomic resources for *Colletotrichumspaethianum* and *Colletotrichumfructicola*

**DOI:** 10.3897/imafungus.16.141732

**Published:** 2025-02-17

**Authors:** Davide D’Angelo, Roberto Sorrentino, Tiphany Nkomo, Xianzhi Zhou, Niloofar Vaghefi, Byron Sonnekus, Tanay Bose, Domenico Cerrato, Loredana Cozzolino, Nicky Creux, Nunzio D’Agostino, Gerda Fourie, Giovanna Fusco, Almuth Hammerbacher, Alexander Idnurm, Levente Kiss, Yanping Hu, Hongli Hu, Ernesto Lahoz, Jason Risteski, Emma T. Steenkamp, Maurizio Viscardi, Magriet A. van der Nest, Yuan Wu, Hao Yu, Jianjin Zhou, Chinthani S. Karandeni Dewage, Loly I. Kotta-Loizou, Henrik U. Stotz, Bruce D. L. Fitt, Yongju Huang, Brenda D. Wingfield

**Affiliations:** 1 Department of Agricultural Sciences, University of Naples Federico II, piazza Carlo di Borbone 1, 80055, Portici, Naples, Italy; 2 Research Centre for Cereal and Industrial Crops (CREA-CI), via Torrino 3, 81100, Caserta, Italy; 3 Department of Biochemistry, Genetics and Microbiology, Forestry and Agricultural Biotechnology Institute (FABI), University of Pretoria, Pretoria, 0028, South Africa; 4 Institute of Plant Protection, Fujian Academy of Agricultural Sciences, Wusi Road 247, Fuzhou 350003, China; 5 School of Agriculture, Food and Ecosystem Sciences, Faculty of Science, The University of Melbourne, Parkville, Australia; 6 Department of Zoology and Entomology, Forestry and Agricultural Biotechnology Institute (FABI), University of Pretoria, Pretoria, 0028, South Africa; 7 Istituto Zooprofilattico Sperimentale del Mezzogiorno, Via Salute 2, 80055, Portici, Naples, Italy; 8 Department of Plant and Soil Science, Forestry and Agricultural Biotechnology (FABI), University of Pretoria, Pretoria 0028, South Africa; 9 School of BioSciences, Faculty of Science, The University of Melbourne, Parkville, Australia; 10 Centre for Crop Health, University of Southern Queensland, Toowoomba, Australia; 11 Eszterházy Károly Catholic University, Eger, Hungary; 12 Plant Protection Institute, Centre for Agricultural Research, HUN-REN, Budapest, Hungary; 13 College of Plant Protection, Fujian Agriculture and Forestry University, Fuzhou 350002, China; 14 Hans Merensky Chair in Avocado Research, Forestry and Agricultural Biotechnology Institute (FABI), University of Pretoria, Pretoria 0028, South Africa; 15 Technology Center, Xiamen Customs, Xiamen 361026, China; 16 Sanming Academy of Agricultural Sciences/Fujian Key Laboratory of Crop Genetic Improvement and Innovative Utilization for Mountain Area, Sanming, Fujian 365051, China; 17 Centre for Agriculture, Food and Environmental Management Research, School of Life and Medical Sciences, University of Hertfordshire, Hatfield, Hertfordshire, AL10 9AB, UK

## ﻿IMA GENOME-F 20A A draft genome assembly of *Agroatheliarolfsii*, the aetiological agent of southern blight disease on industrial hemp

### ﻿Introduction

Hemp is an ancient plant that, over the centuries, has united generations and continents, shaping agricultural landscapes. Over time, the economies, uses and traditions of many people have been influenced by this species and its numerous applications (Clarke and Merlin 2016).

Italy, a major producer and exporter of hemp, has progressively reduced its cultivated area to the point of almost eliminating it due to the laboriousness of cultivation and processing, the diffusion of synthetic fibres, the availability of alternative and cheaper vegetable fibres such as cotton and jute, and regulations on the use of drugs (Amaducci et al. 2015).

With the approval of Law N. 242 of 2 December 2016, entitled “Provisions for the promotion of hemp cultivation and the agro-industrial supply chain,” the interest of the agricultural, industrial, and entrepreneurial world towards hemp and its multifunctionality is renewed.

Hemp is susceptible to several diseases (Sorrentino et al. 2019). Among the plethora of pathogens that pose a serious threat to hemp cultivation (Punja 2021), the soil borne *Agroatheliarolfsii* (*Sclerotiumrolfsii* Sacc.), the agent of southern blight, is one of the most recognized fungal pathogens in southern temperate zones and the tropics, affecting both fiber and seed cultivations (Ferri 1961a). *A.rolfsii* attacks over 500 plant species (Aycock 1966), including both monocotyledons and dicotyledons (Farr et al. 1989). Attacks on hemp have been reported in India, the United States, Japan, Greece, and Italy (Ferri 1961a; McPartland and Clarke 2000; Amaradasa et al. 2020; Mersha et al. 2020; Chatzaki et al. 2022).

Fungi of the genus *Sclerotium* are characterized by the production of sclerotia and sterile mycelia, devoid of spores. The genus *Sclerotium* includes more than 40 phytopathogenic species (Farr 2008).

Many *Sclerotium* species reproduce asexually, with sexual reproduction rare, and are recognized exclusively in their anamorphic phase (Punja and Rahe 2001). *Sclerotiumrolfsii*, the best-known species of the genus, presents the teleomorph phase *Agroatheliarolfsii* (Curzi), characterized by the development of resupinate basidiocarps and hyphal filaments emerging from the germinating sclerotia (Tu and Kimbrough 1978).

A total of three *A.rolfsii* isolates, which produce symptoms of stem rot disease on groundnut, were isolated. Their genomes were sequenced and assembled, providing insights into aspects of the pathogen’s pathogenicity (Iquebal et al. 2021; Yan et al. 2021). Iquebal et al. (2021) released a genome (MR10 strain) of 72.3 Mb in length and identified key virulence genes. Yan et al. (2021) compared the genomes of two different strains, namely GP3 and ZY, and revealed variations in their pathogenicity associated with a significant difference in aggressiveness on peanut plants.

**Figure 1.**
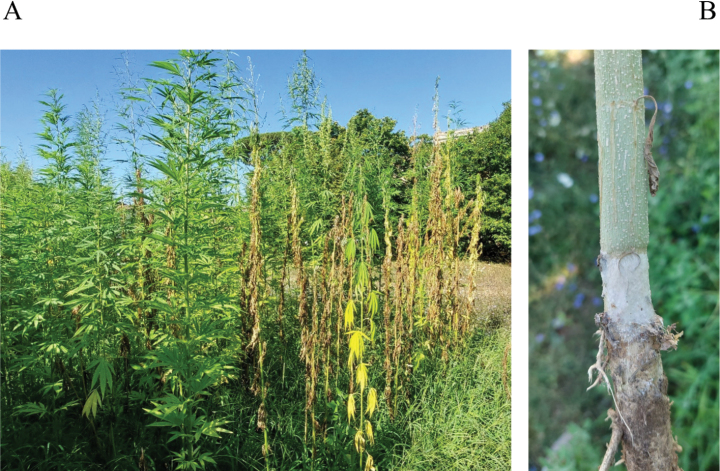
**A** Hempseed field where *Agroatheliarolfsii* was discovered, showing symptoms of yellowing and desiccation **B** hemp plant collar where the pathogen creates a constriction with prolific production of both mycelium and sclerotia.

Noteworthy, strains MR10 and ZY, which have genome sizes exceeding 70 Mb, are noted in the NCBI database as “atypical assemblies” due to their unusually large genome sizes. Assembling and annotating the genome of *A.rolfsii* isolated from hemp can help researchers to develop more effective control strategies to protect this crop. The identification of putative virulence candidate genes can be exploited to develop agrochemicals or targeted therapeutic approaches for disease control, mainly in the post-penetration phase. Here, we report the draft genome of *A.rolfsii* isolated from industrial hemp in Campania in June 2022 (Fig. 1).

### ﻿Sequenced strain

Italy: Campania: Caserta. Isolated from symptomatic plant collar of *Cannabissativa* L. var. Codimodo, 07/2021.

### ﻿Nucleotide sequence accession number

The draft genome sequence has been deposited in NCBI GenBank under the accession number JAXRUS000000000. The version described in this paper is version JAXRUS010000000. Accession for SRA data: PRJNA1052013.

### ﻿Fungal isolation

The collars of 10 symptomatic plants were washed under running water for 10 minutes, then cut into 3 mm sections. For each sample, five sections of the collar underwent surface sterilization by immersion in 5% sodium hypochlorite for 1 minute, followed by immersion in 70% ethanol for an additional minute. The excised small pieces of tissue were then rinsed five times in sterile distilled water in a 50 ml tube, dried by blotting on sterile 3 mm Whatman paper, subdivided into smaller portions of tissue and, finally plated on potato dextrose agar (PDA) amended with 100 mg liter^−1^ of streptomycin sulphate and stored at 24 °C ±2 °C in the dark. Plates were inspected daily, and growing colonies were sub-cultured on PDA for 10 days. In total, 50 monohyphal purified fungal isolates were obtained. Colonies of these isolates were white and produced sclerotia after 10 days. We identified the target fungal isolate from the pool by sequencing the Internal Transcribed Spacer (ITS), as amplified using PCR. DNA was extracted using the DNeasy Plant Mini Kit (Qiagen, USA) following the manufacturer’s instructions and PCR reactions were carried out using 10 ng of DNA with the ITS1/ITS4 primers (White et al. 1990) specific for the ITS regions. Each 50 μL reaction mixture included 1X reaction buffer, 200 μM dNTPs, 500 mM of each primer, and 1 unit of Q5 Hi-Fi DNA polymerase (New England Biolabs, UK). The PCR conditions were the following: denaturation at 94 °C for 1 minute, 35 cycles of 94 °C for 30 seconds, 58 °C for 30 seconds, and 72 °C for 30 seconds, with a final extension at 72 °C for 5 minutes. Amplicons were purified using the Nucleospin Extract Kit (Macherey–Nagel, Germany) and sequenced at BMR Genomics (Padova-Italy). Fungi are maintained in PDA slant tubes and in a 20% glycerol stock solution at the phytopathology laboratory of the CREA, Cereal and Industrial Crop Research Centre, Caserta.

### ﻿DNA isolation and sequencing library preparation

DNA was purified from mycelia obtained by culturing *A.rolfsii* on PDA plates covered with cellophane films and kept in the dark at 24 °C for 7 days. Mycelia were collected, ground in liquid nitrogen and 100 mg were used to isolate the DNA using the QIAGEN® Genomic-tip kit following the manufacturer’s instructions. Genomic DNA was resuspended in EB buffer (Qiagen) to avoid EDTA in the final preparation. DNA quality (260/280 ratio) was assessed using a NanoDrop ND-1000 UV-Vis Spectrophotometer (NanoDrop Technologies Inc., Wilmington, DE). The amount of DNA was assessed using the Qubit platform (Invitrogen, Paisley, UK). Library preparation was performed using ILMN DNA LP (M) Tagmentation (96 Samples, IPB), and IDT® for Illumina® DNA/RNA UD Indexes Set A, Tagmentation (Illumina Italy S.r.l. Milano-Italy) according to the Illumina DNA preparation guide. The quality and amplicon size of the libraries were assessed using a Tapestation 4150 (Agilent) at multiple steps during the protocol, typically after size selection and PCR amplification. Three different libraries were normalized to a working concentration pool of 12 pM using the molarity calculated from Qubit measurements adjusted for fragment size. The sequencing run was performed on Illumina MiSeq (Illumina), equipped with MiSeq Reagent Kit v3 (600 cycles) which includes: Paired-End Reagent Plate, MiSeq Flow Cell.

### ﻿Read pre-processing

Quality control on FASTQ files (paired-end 150 bp) was performed using FastQC v0.11.9 (Andrews 2020). The raw reads were subjected to a pre-processing step using Trimmomatic v0.39 (Bolger et al. 2014) with the following parameters: SLIDINGWINDOW:4:20 LEADING:20 TRAILING:20 MINLEN:75. High quality reads were used to compute the *k*-mer distribution using Kmergenie v1.7051 (Chikhi and Medvedev 2014). Genome size was estimated using GenomeScope v1.0 (Vurture et al. 2017) based on the *k*-mer distribution.

### ﻿Genome assembly

*De novo* genome assembly was performed using ABYSS v2.3.5 (Jackman et al. 2017), following the default pipeline for paired-end libraries. The *k*-mer size was set to 107 bp. Scaffolding was performed with Ragtag v2.1.0 (Alonge et al. 2022), sorting and orienting contigs along the *A.rolfsii* reference genome (GenBank accession number GCA_018343915.1) using a whole-genome alignment approach.

Unique alignments less than 500 bp and scaffolds less than 1,000 bp were filtered out. Gap closure was achieved using Sealer v2.3.5 (Paulino et al. 2015), employing the initial reads and considering 150 bp-sized “flanks” of the scaffolds as pseudo-reads.

Finally, the draft genome sequence was subjected to repeat region masking using RepeatMasker v4.1.2 (Tarailo-Graovac and Chen 2009) in slow search mode with *rmblastn* as the search engine and the *Agaricomycetidae* dataset from the Dfam repository (Storer et al. 2021) release 3.3 as filtering database.

### ﻿Assembly assessment

Quality assessment of the assembly was performed with QUAST v5.2.0 (Gurevich et al. 2013). Assembly completeness was assessed by mapping reads to the final assembly using BWA v0.7.17 (Li and Durbin 2009) with default parameters. The completeness of the genome annotation was assessed based on BUSCO v5.4.4 (Simão et al. 2015) using *Agaricomycetes* as the reference dataset.

### ﻿Comparative and phylogenetic analysis

The assembled genome was compared with the *A.rolfsii* reference genome downloaded from GenBank (accession number GCA_018343915.1) using MUMmer v3.23 (Marçais et al. 2018), performing a whole genome alignment with a minimum cluster length of 100.

The ITS sequence from an *A.rolfsii* strain isolated from *C.sativa* (GenBank accession: MZ242252.1) was utilized as a BLASTn query to identify the corresponding ITS sequence within the assembled genome. A phylogenetic analysis was then conducted using the identified ITS sequence from the draft genome, along with sequences from 16 *A.rolfsii* strains, 5 *A.delphinii* strains, 3 *A.coffeicola* strains, and a *Rhizoctoniasolani* strain used as an outgroup. Multiple sequence alignment was performed using Mafft v.7.490 with the G-INS-i method and 1,000 iterations. Finally, a phylogenetic tree was generated using IQ-TREE v2.0.7 (Minh et al. 2020) using the ModelFinder option to select the best DNA substitution model and performing 1,000 ultrafast bootstrap replicates for the consensus tree inference. The resulting tree was visualized and edited using the ITOL tool v5 (Letunic and Bork 2021).

The protein complement of *A.rolfsii* was compared with the proteomes of four other fungal species belonging to the *Agaricomycotina*. To this end, the protein sequences of *Rhizoctoniasolani* (GCA_016906535.1), *Armillariamellea* (GCA_030407055.1), *Agaricusbisporus* (GCA_000300575.1), and *Pleurotusostreatus* (GCA_014466165.1) were downloaded from GenBank.

OrthoFinder v2.5.5 (Emms and Kelly 2018) was used with default settings to identify orthologous groups. The ggVennDiagram v1.4.9 R package (Gao et al. 2021) was used to create a Venn diagram showing the number of shared orthologous groups.

BinGO v3.0.3 software (Maere et al. 2005) was used to perform GO enrichment analysis. GO terms associated with *A.rolfsii* species-specific proteins were used as the test set, while GO terms associated with the complete set of proteins were selected as the background set.

The enrichment analysis involved a hypergeometric test and a Benjamini and Hochberg false discovery rate (FDR) correction of the p-value. Only GO terms in the biological process and molecular function domains with an FDR-corrected p-value > 0.05 were considered statistically significant.

### ﻿Gene prediction

Gene prediction was performed by running Helixer v0.3.2 (Stiehler et al. 2021), which employs a deep neural network model trained on 128 fungal species and validated on 170 species. Then, the evidence collected from the first round of annotation was used as input for GeneMark-ES v4 (Borodovsky and Lomsadze 2011) with default parameters. Gffread v0.12.8 (Pertea and Pertea 2020) was used to extract coding sequences (CDS) and protein sequences, filtering out those with incorrect start and stop codons.

### ﻿Functional annotation

Blast2GO v3.1.9 (Gotz et al. 2008) was used to assign a functional annotation to the predicted genes. Initially, InterProScan was used to query all InterPro (Paysab-Lafosse et al. 2023) member databases, subsequentially a second annotation run was performed via the EggNOG Mapper tool in BLAST2GO, searching for Cluster of Orthologous Groups (COG). The final gene ontology (GO) annotation was generated by a consensus of the results from both previous analyses. The KAAS web server (Moriya et al. 2007) was used to assign genes to KEGG molecular pathways. Carbohydrate active enzymes (CAZymes) were annotated using the dbCAN3 server (Zheng et al. 2023), to query the dbCAN, dbCANsub, and CAZY databases. A consensus on the three results led to the final list of CAZymes. Proteins putatively involved in host-pathogen interactions were identified by querying the HPIDB 3.0 database (Ammari et al. 2016) limiting the search to the model species *Arabidopsisthaliana* and *Saccharomycescerevisiae*. Effector proteins were predicted using the Phobius web server (Käll et al. 2007), retaining only proteins with signal peptides and no transmembrane domains. Sequences with a maximum length of 500 amino acids and at least 5 cysteine residues were retained and collected into the list of high-fidelity effectors. Finally, gene sequences were submitted to the antiSMASH-fungal web server v6.0 (Blin et al. 2023) to identify gene clusters involved in the synthesis of secondary metabolites.

### ﻿Results and discussion

The sequencing run yielded 10.5 Gbp with a Q index >30. A total of 25,898,770 high-quality paired-end reads were used for genome assembly, resulting in 3,138 contigs, whose total sequence length is 44,845,534 bp with an N50 of 3,998,658 bp (Table 1).

The size of the assembled genome were larger than that estimated based on *k*-mer distribution (~45 Mb vs ~37 Mb) assuming a genome coverage of 86.6 X. A total of 427,196 bp (0.95%) were masked, comprising 9,616 simple repeats and 1,663 low complexity regions. The reads used in the assembly process were mapped onto the assembled genome to assess assembly accuracy. Over 99.9% of the reads were successfully mapped.

A total of 14,804 genes was predicted, including 11,230 protein-coding genes and 3,574 non-coding RNAs. The BUSCO analysis indicated a completeness level of 92.5% for the *Agaricomycetes*, identifying 2,471 complete and single-copy BUSCOs, along with 208 complete but duplicated BUSCOs, and 72 fragmented BUSCOs. A total of 36,139 GO terms were assigned to 4,428 protein coding genes. A total of 4,086 proteins has been assigned to KEGG, covering 311 pathways (Suppl. material 1). Functional annotation has allowed the identification of various genes that may be involved in pathogenesis processes and could be potential targets for phenotyping studies. Within the annotated proteins, 272 putative carbohydrate-active enzymes were identified (Suppl. material 1).

The annotation of genes involved in the biosynthesis of secondary metabolites led to the identification of 137 genes grouped into 36 clusters (Suppl. material 1).

Nine hundred sixty-two (N. = 962) proteins possibly secreted into the extracellular space were identified, as they include a signal peptide and lack transmembrane domains. Further filtering based on the number of amino acid residues and cysteine content reduced the dataset to 30 putative pathogenesis-related (PR) proteins (Suppl. material 1).

Finally, exploration of plant-pathogen interactions led to the identification of 73 protein-coding genes involved in 126 interactions, of these, 37 were classified as “association”, 26 as “direct interaction”, 31 as “physical association”, 29 as “suppressive genetic interaction defined by inequality” and one as “colocalization” (Suppl. material 1).

**Figure 2.**
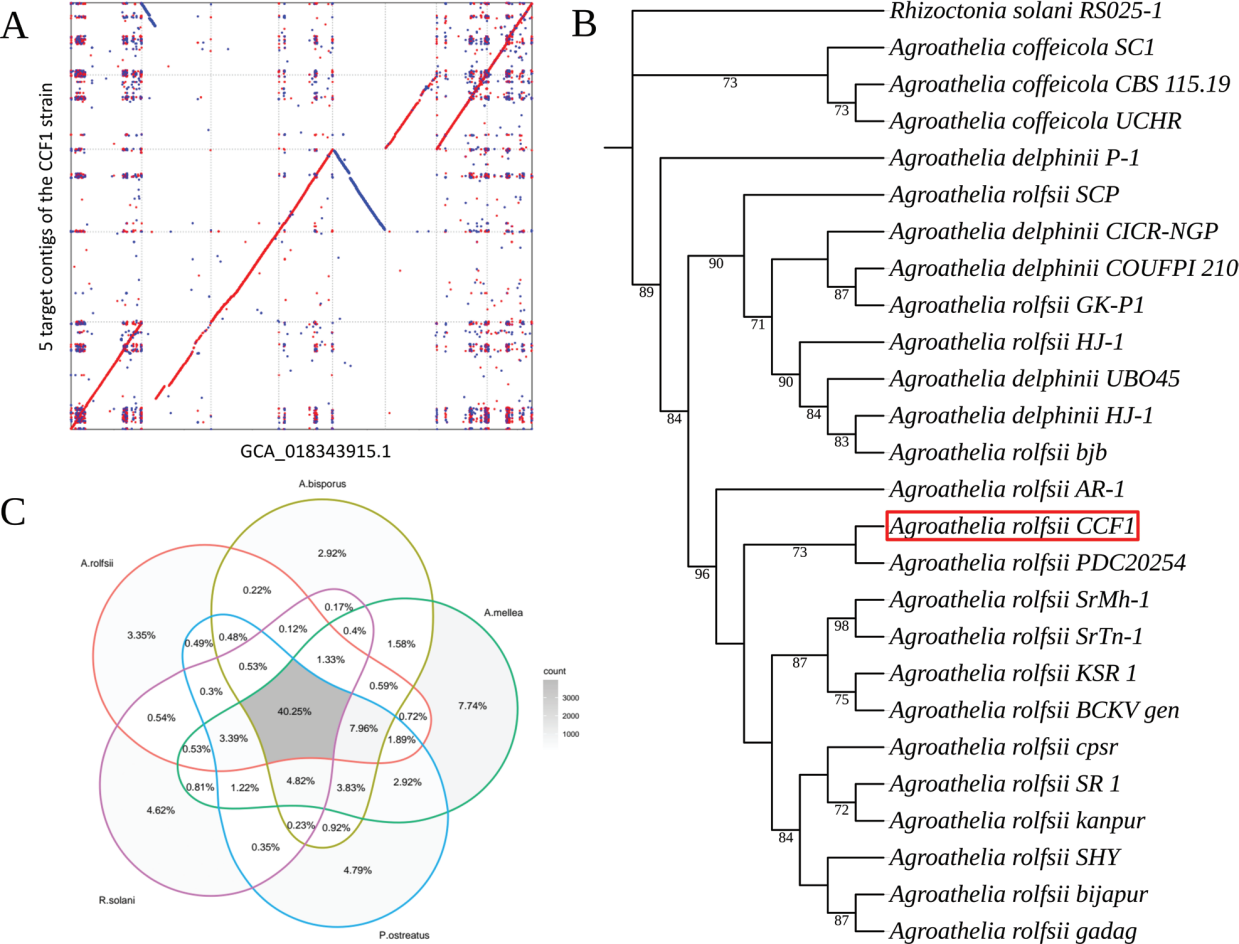
**A** Dot plot illustrating the alignment results between the five largest contigs assembled in this study and the 8 scaffolds from the *A.rolfsii* reference genome (GenBank accession: GCA_018343915.1) **B** Maximum-Likelihood tree constructed using Internal Transcribed Spacer (ITS) sequences, depicting the phylogenetic relationships among taxa within the *Agroathelia* genus. *Rhizoctoniasolani* was used as the outgroup, with bootstrap values > 70 indicated on the branches **C** Venn diagram displaying orthogroups identified in the proteomes of *A.rolfsii* and four other fungal species within the *Agaricomycotina*.

Alignment between the 3,138 contigs and the 8 scaffolds of the *A.rolfsii* reference genome (GCA_018343915.1) resulted in 76.34% aligned bases and a total of 584,388 single nucleotide polymorphisms, indicating a high degree of similarity. Furthermore, aligning only the five largest contigs still provided good coverage of the GCA_018343915.1 reference genome (Fig. 2A).

The substitution model “K3Pu+F” was identified as the most suitable model for tree inference using the maximum likelihood method. The ITS sequence-based tree positioned the *A.rolfsii* strain CCF1 within a major clade alongside other *A.rolfsii* strains, showing a distinct clustering pattern. In contrast, *A.delphinii* and *A.coffeicola* strains formed separate clusters (Fig. 2B).

Comparison between the proteomes of *A.rolfsii*, *R.solani*, *A.mellea*, *A.bisporus* and *P.ostreatus* led to the identification of 9,854 orthogroups, 3,967 (40.3%) of which were classified as core-orthogroups. A total of 10,441 (93%) *A.rolfsii* protein-coding genes were assigned to 6,176 orthogroups, of which 330 (3.35%) were species-specific (Fig. 2C). The latter contained 1,916 species-specific genes, which were subjected to Gene Ontology enrichment analysis, revealing 42 overrepresented GO terms in the biological process domain including some of the most significant ones related to macromolecules and secondary metabolite biosynthetic processes, responses to environmental stresses, biosynthesis of aromatic compounds, defence responses to other organisms.

Plant pathogenic fungi pose a major threat to agriculture, causing significant yield losses. The development of new organic or traditional agrochemicals and targeted therapeutic approaches for the control of plant pathogenic fungi requires a comprehensive knowledge of their gene repertoire.

In the present study, the genome of the pathogenic fungus *A.rolfsii* strain CCF1 isolated from hemp (*Cannabissativa* L.) was assembled and annotated. The data produced will allow the scientific community to have a further resource to study genetic architecture and discover new effectors and their pathogenic mechanism. Indeed, few *A.rolfsii* genomes have been assembled so far. Although fragmented into over 3,000 contigs, the genome we released is quite complete. Indeed, the five largest contigs cover much of the genome of another *A.rolfsii* strain sequenced using third-generation sequencing technologies (Yan et al. 2021).

In this study, a combination of self-learning and deep learning algorithms, so far rarely applied in fungal genome annotation, allowed the prediction of a considerable number of genes: 11,230 protein-coding genes and 3,574 non-coding RNAs. The quality of the annotation was satisfactory and near-complete as 92.5% of the *Agaricomycetes* BUSCOs were identified.

The phylogenetic analysis revealed the evolutionary context of *A.rolfsii* CCF1, placing it within a major clade that exclusively includes *A.rolfsii* isolates. In contrast, *A.coffeicola* and *A.delphinii* isolates were positioned in separate clades. The search for orthologs between different fungal species led to the identification of core-orthogroups, thus highlighting common and species-specific protein-coding genes.

The identification of overrepresented GO terms associated with species-specific genes provides valuable insights into the molecular mechanisms that have shaped the biology of *A.rolfsii* CCF1. The presence of these genes suggests evolutionary innovations that have contributed to its divergence and adaptation from other fungal relatives within the *Agaricomycotina*. Studying these genetic features can enhance our understanding of the evolutionary processes driving diversification and specialization among fungi. From a biological perspective, analysing species-specific proteins in *A.rolfsii* through GO enrichment provides insights into unique biological processes and molecular functions critical for its survival and adaptation. The presence of various types of interactions suggests a complex relationship between the fungal pathogen and the host plant; these could be studied to gain a deeper understanding of the molecular mechanisms of host-pathogen coevolution and adaptation. In addition, the effector identification procedure narrows the field to a small number of target genes for further study. Further studies on these genes could deepen our knowledge of *A.rolfsii*’s biology, illuminate its distinct characteristics, and aid in developing precise strategies for managing hemp diseases.

**Authors**: Davide D’Angelo, Roberto Sorrentino, Ernesto Lahoz, Domenico Cerrato, Maurizio Viscardi, Loredana Cozzolino, Giovanna Fusco, Nunzio D’Agostino*

*Contact: nunzio.dagostino@unina.it

**Table 1.** Summary statistics on genome assembly.

Assembly features	*A.rolfsii* scaffolds
Total sequence length (bp)	44,845,534
Number of contigs	3,138
Number of contigs (≥50,000 bp)	16
Largest contig size (bp)	5,959,244
GC content (%)	46.49
N50	3,998,658
N90	4,804
L50	5
L90	1,500
Missingness (%)	0.406

**Figure 3.**
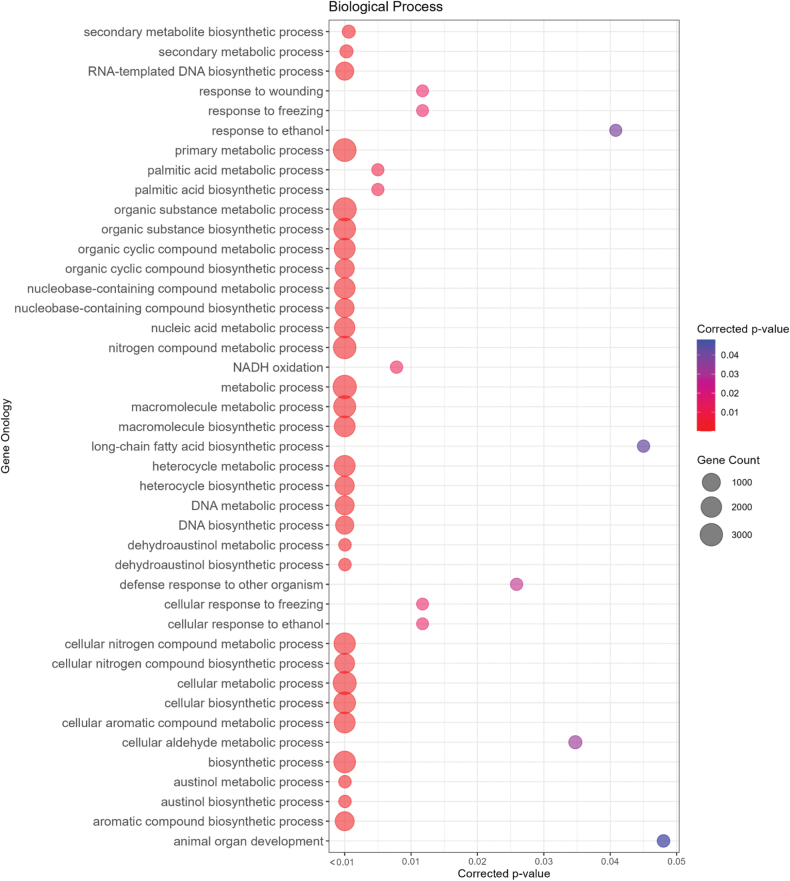
Enriched gene ontology terms in the Biological Processes domain were identified for *A.rolfsii* species-specific protein-coding genes following the OrthoFinder analysis.

## ﻿IMA GENOME-F 20B Draft genome assembly of *Ceratobasidiumpapillatum* CBS 570.83

### ﻿Introduction

*Ceratobasidium* is a genus of *Basidiomycetes* fungus that plays diverse ecological roles (Mosquera-Espinosa et al. 2013). Many *Ceratobasidium* species are plant pathogens, such as *Ceratobasidiumtheobromae*, *C.cereale*, *C.cornigerum*, and *C.anceps*, which cause diseases like root rot, white thread blight, vascular streak dieback, and damping-off (Schroeder and Paulitz 2012; McMahon and Purwantara 2016; De Melo et al. 2018; Lima et al. 2020). In contrast some species, such as *C.bicorne* and *C.cornigerum*, are saprophytes with bioremediation potential (Fazary et al. 2016; Harrington et al. 2011; Haas et al. 2008). *Ceratobasidium* species are considered *Rhizoctonia*-like fungi that form symbiotic associations with orchids (Warcup and Talbot 1980; Dearnaley 2007; Decruse et al. 2018; Thixton et al. 2020).

Presently, there are 44 species within the *Ceratobasidium* genus (https://www.mycobank.org/). However, DNA sequences are unavailable for the majority of these species. Genomic data are only available for *C.theobromae*, the causative agent of vascular-streak dieback (VSD) in cacao (Ali et al. 2019). While other *Ceratobasidium* genomes are available on NCBI and MycoCosm, they pertain to undescribed species. To fill this gap, we sequenced, assembled, and annotated the genome of *C.papillatum*.

*Ceratobasidiumpapillatum* was described as a symbiont of orchids by Warcup and Talbot (1980). It has subsequently been detected in the roots of various other orchids (Freestone et al. 2021). Thus, the genome sequence of this species is a pivotal foundation for conducting mechanistic and comparative studies, encompassing both beneficial and pathogenic fungi within the *Ceratobasidium* genus.

### ﻿Sequenced strain

Australia: Toowoomba, Queensland: isolated from *Sarcochilusdilatatus* (CBS 570.83 ex type strain).

### ﻿Nucleotide sequence accession number

This Whole Genome Shotgun project for *Ceratobasidiumpapillatum* CBS 570.83 has been deposited at DDBJ/ENA/GenBank under the accession JAYRCO000000000.1; BioProject PRJNA1046290 and BioSample SAMN38480858. The version described in this paper is version JAYRCO010000000.

### ﻿Materials and methods

The isolate *Ceratobasidiumpapillatum* (CBS 570.83) was received from the Westerdijk Fungal Biodiversity Institute in the Netherlands. The isolate was revitalised on half-strength potato dextrose agar (PDA) medium (19 g PDA powder (Merck, South Africa); 7 g agar; distilled water 1 L) at 25 °C. Total genomic DNA was extracted from 14-day-old cultures following the protocol suggested by Duong et al. (2013).

The sequencing library was prepared using the MGIEasy Universal DNA library prep kit to generate 150 bp paired-end libraries. Whole genome sequencing was performed using the MGI DNBSeq G400 sequencer at the Agricultural Research Council Biotechnology Platform, South Africa (ARC-BTP) using the PE150 sequencing strategy.

The quality of the reads was assessed using FastQC v0.11.7 (https://github.com/s-andrews/FastQC) and was assembled using SPAdes v3.15.0 (Bankevich et al. 2012). Quast v5.0.2 (Mikheenko et al. 2018) was used to summarize the genome statistics. The completeness of the genome assembly was determined with BUSCO v5.3.2 utilizing the fungi*_odb10*, Basidiomycota*_odb10* and Agaricomycetes*_odb10* lineage datasets (Manni et al. 2021b). The Funannotate v1.8.15 (https://github.com/nextgenusfs/funannotate) with default settings was used to annotate the assembled genome.

To confirm the identity of the isolate used for genome sequencing, phylogenetic analysis was conducted using maximum likelihood (ML) and Bayesian (BI) approaches. For this, a sequence dataset was prepared that included the complete internal transcribed spacer (ITS) gene region extracted from the assembled genome using CLC Genomics Workbench v23.0.5 (CLC bio, Aarhus, Denmark) and 16 *Ceratobasidium* species retrieved from NCBI GenBank, including the ITS sequence of the same *C.papillatum* isolate deposited by CBS (NR_154600). *Cantharelluseucalyptorum* (JN944001) was used as an outgroup. This dataset was aligned using MAFFT v7.490 (Rozewicki et al. 2019) using default settings and manually adjusted using Mesquite v3.81 (https://www.mesquiteproject.org/). Phylogenetic analysis using ML was performed with IQ-TREE v1.2 (Trifinopoulos et al. 2016) using 1,000 bootstrap replicates. BI analysis was performed using MrBayes v3.2.7 (Ronquist et al. 2012) with four MCMC chains initiated from a random starting tree and run for 5 million generations. The stop value was set at 0.01, the temperature at 0.2 and trees were sampled every 100 generations. A burn-in of 30% of the sampled trees was discarded, and the remainder were used to generate majority rule consensus trees. The phylogenetic trees were viewed and rooted using FigTree v1.4.4 (http://tree.bio.ed.ac.uk/software/figtree/).

### ﻿Results and discussion

**Figure 4.**
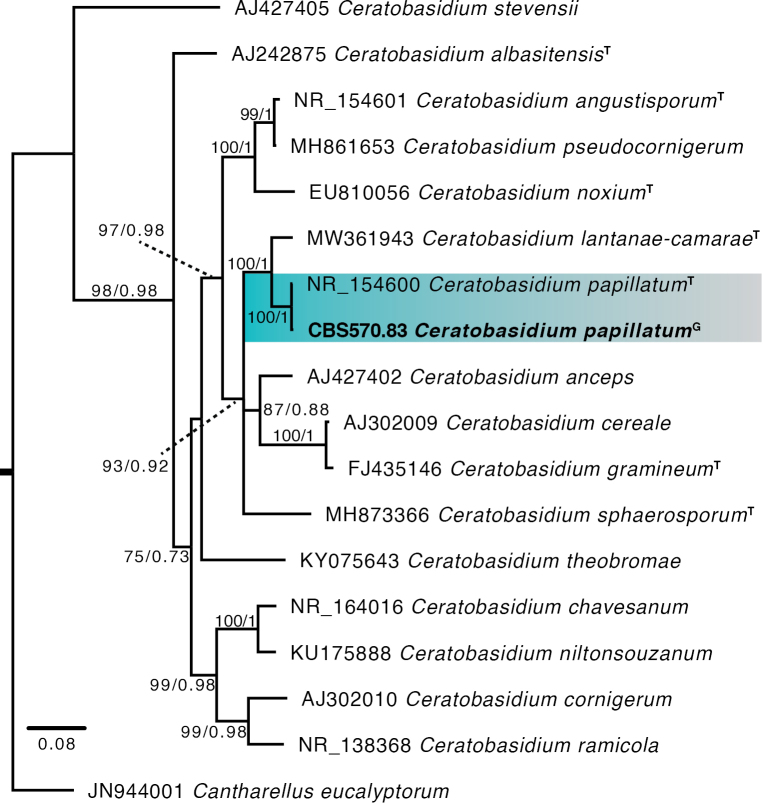
The maximum likelihood tree constructed using ITS sequences of 16 *Ceratobasidium* species. Type isolates and the sequence extracted from the genome of *Ceratobasidiumpapillatum* (CBS 570.83) are suffixed with T and G, respectively. *Cantharelluseucalyptorum* served as an outgroup. Branch support values are indicated as bootstrap values/posterior probabilities. Support values below 70 and 0.80 were deemed unreliable and, hence, were excluded from the tree.

The *C.papillatum* genome was 41.00 Mb in size and assembled into 3,091 contigs, of which 2,897 were longer than 1,000 bp. The N50 and N75 values were 23,435 bp and 12,862 bp, respectively, while the L50 and L75 values were 520 and 1,119, respectively. The GC content was 49.01%. Apart from *C.papillatum*, only one genome from a well-described *Ceratobasidium* species, *C.theobromae*, is available in GenBank and Mycocosm. However, genomes from several undescribed *Ceratobasidium* species are also present in these databases. The genome size of *C.papillatum* is 10 Mb larger than that of *C.theobromae*, yet 17–90 Mb smaller than those of the undescribed species. This shows a notable variation in the genome sizes of *Ceratobasidium* species. BUSCO analysis showed completeness of 93.5%, 92.5% and 79.3% using the *Fungi*, *Basidiomycota* and *Agaricomycetes* datasets, respectively. Notably, the number of predicted genes for *C.papillatum* (12,614) was found to be smaller than that of other *Ceratobasidium* species, indicative of the smaller genome size. The phylogenetic position of this isolate, using the genomic copy of the ITS region, confirms that the sequenced genome is *C.papillatum* (Fig. 4). This genome provides a valuable resource for future comparative studies and in-depth analysis of genome structure within the genus. Thus, it will address fundamental questions involving the biology and lifestyle of this group of fungi.

**Authors**: Tiphany Nkomo, Almuth Hammerbacher*, Tanay Bose, Brenda D. Wingfield*

*Contact: Brenda.Wingfield@fabi.up.ac.za; Almuth.Hammerbacher@fabi.up.ac.za

## ﻿IMA GENOME-F 20C Genomic resources for *Colletotrichumspaethianum* and *Colletotrichumfructicola*, causal agents of Anthracnose in *Polygonatumcyrtonema*

### ﻿Introduction

*Polygonatumcyrtonema* Hua. (Duohua Huangjing in Chinese; family *Asparagaceae*), is an edible medicinal plant widely used in China to treat diabetes and asthma (Pharmacopeia Committee of P. R. China, 2020). The rhizomes are particularly valued as the primary components in various medicines and food. However, wild *P.cyrtonema* resources cannot meet the increasing demand for health products, such as Huangjing liquor, Huangjing tea, Huangjing noodles, etc., making artificial cultivation a necessity. Unfortunately, many diseases also appeared as amplified with the development of artificial cultivation of *P.cyrtonema*. Anthracnose is one of the common diseases found on *P.cyrtonema* and has become a serious threat to *P.cyrtonema* production. The leaf and stem symptoms include large elliptical, irregular or long strip spots, with pale to dark brown lesions, in some case intermediate white necrosis (Zhou et al. 2017; Ma et al. 2020; Dan et al. 2023). On the fruit, dark brown necrotic lesions are usually observed. In the major growing regions of Guangze County, Nanping City (northern Fujian Province, China), anthracnose incidence reaches approximately 20% during the flowering stage. As temperature and humidity increase, anthracnose incidence in some areas can rise to over 50% during the fruit stage (Zhou et al. 2017).

*Colletotrichum* is the pathogen responsible for many plant anthracnose and among the most important plant pathogenic fungi worldwide (Dean et al. 2012). For example, *C.spaethianum* is a well-known pathogen of anthracnose in plants belonging to the genus *Polygonatum* (Tomioka et al. 2008; Liu et al. 2020; Ma et al. 2021; Utami and Hiruma 2022). Another member of this genus, *C.fructicola*, has a broad host range, including tobacco, apples, strawberries, orchids, citrus, tea-oil trees, *Parispolyphylla*, etc (Li et al. 2016; Wang et al. 2016; Hu et al. 2019; Silva-Cabral et al. 2019; Liang et al. 2020; Zhou et al. 2020). Thus far, one *C.spaethianum* (MAFF 239500) genome (Utami and Hiruma 2022) and 16 *C.fructicola* genomes have been deposited in NCBI genome database (https://www.ncbi.nlm.nih.gov/genome/); *C.fructicola* Nara and *C.fructicola* CGMCC3.1737, strawberry anthracnose pathogens (Gan et al. 2013; Armitage et al. 2020); isolates 15060 and 1104-7 of *C.fructicola*, causal agents of mango (Li et al. 2018) and apple (Liang et al. 2018; Liang et al. 2020) anthracnose.

Nanping is an important *P.cyrtonema*-producing region with a total planting area of approximately 1,000 ha. Thus, identifying the *Colletotrichum* species causing *P.cyrtonema* anthracnose in Nanping is a key issue. Anthracnose-diseased samples of *P.cyrtonema* were collected from four cultivation areas in Nanping City, Fujian Province: Chongren, Yushan, Heping, and Huaqiao. Previously, 76 representative *Colletotrichum* strains were isolated from diseased samples based on morphological characteristics and multilocus phylogenetic analysis. 13 species were identified, with two being novel, and then 12 were first reported in *P.cyrtonema*. The dominant causal agents of anthracnose in *P.cyrtonema* were *C.spaethianum* (31 strains, 40.79%) and *C.fructicola* (20 strains, 26.32%). *C.spaethianum* infects leaves, stems, and fruit stalks, while *C.fructicola* infects leaves, stems, and fruits. The former species is more serious leaves, while the latter is more virulent on stems. So far, the genetic differences behind these species are not clear as there are no available genome resource. Here, we report the genome of one isolate of *C.spaethianum* Y1_DY3_A (hereafter CsY1), and one isolate of *C.fructicola* C1_DY2_B (hereafter CfC1) from *P.cyrtonema*.

### ﻿Sequenced strains

*Colletotrichumspaethianum*: **China**: Fujian: Huaqiao, isolated from infected leaves of *P.cyrtonema*, 2021, X. Zhou (Y1_DY3_A).

*Colletotrichumfructicola*: **China**: Fujian: Chongren, isolated from infected leaves of *P.cyrtonema*, 2021, X. Zhou (C1_DY2_B).

### ﻿Nucleotide sequence accession number

All reported genome sequences and genes have been deposited in the Genome Warehouse (GWH, https://ngdc.cncb.ac.cn/gwh, accession number GWHEQUI00000000 (CfC1) and GWHEQUR00000000 (CsY1) at the National Genomics Data Center, China National Center for Bioinformation (CNCB-NGDC Members and Partners 2023). Raw sequence reads are publicly accessible at the Genome Sequence Archive (GSA, https://ngdc.cncb.ac.cn/gsa/, accession number CRA013791) of CNCB-NGDC under the BioProject PRJCA021822.

### ﻿Materials and methods

The isolates of *C.spaethianum* Y1_DY3_A (CsY1) and *C.fructicola* C1_DY2_B (CfC1) were deposited in the College of Plant Protection, Fujian Agriculture and Forestry University. The two strains were subcultured on potato dextrose agar (PDA) with 12 h light / 12 h dark at 25 °C for 4 days. The mycelia were collected by vacuum filtration and ground in liquid nitrogen. Genomic DNA was extracted using a rapid fungal genomic DNA isolation kit (Sangon Biotech, Shanghai, China). The purified DNAs were sent to Beijing Novogene Bioinformatics Technology Co. Ltd for library preparation and genome sequencing.

Genome size was estimated in GenomeScope 2.0 (Ranallo-Benavidez et al. 2020) with a 21 bp *k*-mer size and a haploid model. Draft genomes were assembled in Flye version 2.4.2 (Kolmogorov et al. 2019), base errors (mainly indels and SNPs) in the draft genomes were corrected with NextPolish version 1.4.0 (Hu et al. 2020) for both PacBio long reads and Illumina short reads. Using BUSCO version 5.3.0 to evaluate genome completeness for both strains (Manni et al. 2021b). We also assessed genome completeness using the mapping rate of sequenced reads. Long and short reads were mapped to the repeat-unmasked final genome assembly in minimap2 version 2.21-r1071 (Li 2018) and BWA-MEM2 version 2.2.1 (Jung and Han 2022), respectively. Assembly quality was further estimated in Merqury version 1.3 (Rhie et al. 2020) (k = 17) using PacBio self-corrected reads generated with canu version 2.2 (Koren et al. 2017). Using RepeatMasker version 4.1.2 with the assistance of RepeatModeler version 2.02 (http://www.repeatmasker.org/) for repeat sequences *de novo* annotation.

Protein-coding genes were annotated with BRAKER version 2.1.6 (Bruna et al. 2021). EggNOG-mapper v2 (Cantalapiedra et al. 2021) assisted with whole-genome functional annotation in Pfam, Gene Ontology (GO), Kyoto Encyclopedia of Genes and Genomes (KEGG), and EuKaryotic Orthologous Groups (KOG). Diamond version 2.0.11.149 (Buchfink et al. 2021) was used for fungal functional annotations. Furthermore, we deployed OrthoVenn2 (Xu et al. 2019) for comparative whole-proteome cluster analyses to understand protein distribution patterns.

### ﻿Results and discussion

We obtained 8.51 Gb (CsY1, approximately 152×, N_50_ = 12.30 kb) and 18.81 Gb (CfC1, approximately 336×, N_50_ = 17.73 kb) long reads using the PacBio RSII platform, as well as 4.52 Gb (CsY1, approximately 81×) and 8.90 Gb (CfC1, approximately 149×) 150 bp paired-end (PE150) short reads (fragment size ~350 bp) using the Illumina HiSeq 3000 platform. *K*-mer based genome size estimation yielded expected genome sizes of 55.82 Mb for CfC1 and 52.97 Mb for CsY1. Genome assembling using PacBio long reads followed by 2-round base error correction using both of long reads and Illumina short reads resulted in the final genome assemblies of 56,767,350 bp for strain CfC1 (GC: 53.16%) and 54,491,462 bp for strain CsY1 (GC: 52.42%), representing 99.88% and 97.92% of the estimated genome sizes, respectively (Table 2).

The final CfC1 genome contains 13 contigs, with N_50_ and N_90_ contig lengths of 4,938,816 bp (L50:5) and 4,303,157 bp (L90:9), respectively. Its average contig length is 7,120,155 bp and its maximum contig length is 7,595,691 bp. The final CsY1 genome contains 107 contigs, with N_50_ and N_90_ contig lengths of 1,599,232 bp (L50:12) and 319,619 bp (L90:46), respectively. Its average and maximum contig lengths are 509,266 bp and 4,068,025 bp (Table 2).

The assembled CfC1 genome contains 747 (98.55%) complete (744 single, three duplicated) and one fragmented orthologs when compared against the fungi_odb10 lineage (n = 758), as well as 1,666 (97.66%) complete (1,660 single, six duplicated) and two fragmented orthologs compared against the ascomycota_odb10 lineage (n = 1,706). The assembled CsY1 genome contains 740 (97.63%) complete (733 single, seven duplicated) and one fragmented orthologs compared against the fungi_odb10 lineage (n = 758), as well as 1,667 (97.71%) complete (1,653 single, 14 duplicated) and two fragmented orthologs compared against the ascomycota_odb10 lineage (n = 1,706) (Table 3).

Mapping rates of long reads were 99.36% (1,276,006 / 1,284,226 reads) for CfC1 and 99.19% (1,012,391 / 1,020,624 reads) for CsY1); for short reads, they were 99.92% (59,315,969 / 59,362,650 reads, properly paired: 99.02%) for CfC1 and 99.36% (29,999,011 / 30,191,513 reads, properly paired: 97.96%) for CsY1. Assembly quality was further estimated in Merqury revealed average base error rates of 2.09 × 10^-5^ (QV = 46.81) and 2.31 × 10^-4^ (QV = 36.36) for CfC1 and CsY1, respectively, along with 99.88% and 97.92% genome completeness (Table 3).

We then *de novo* annotated and masked 1,983,026 bp (3.49% of genome size) and 4,111,949 bp (7.55%) of repeat sequences for CfC1 and CsY1, respectively. In CfC1 repeats, 1,372,796 bp (69.23%) were interspersed repeats, including short interspersed nuclear elements (SINEs, 5,009 bp), long interspersed nuclear elements (LINEs, 242,286 bp), long terminal repeats (LTRs, 241,689 bp), DNA transposons (491,988 bp), and Unclassified (391,824 bp). In CsY1 repeats, 3,483,650 bp (84.72%) were interspersed, including SINEs (3,657﻿ bp), LINEs (125,572 bp), LTRs (2,405,105 bp), DNA transposons (148,291 bp) and Unclassified (801,025 bp) (Table 4).

We identified 16,928 and 15,753 protein-coding genes in CfC1 and CsY1, respectively, 11,547 (68.21%, CfC1) and 11,456 (72.72%, CsY1) genes in Pfam, 6,837 (40.39%, CfC1) and 6,778 (43.03%, CsY1) genes in GO, 5,121 (30.25%, CfC1) and 6,428 (40.80%, CsY1) in KEGG, as well as 12,378 (73.12%, CfC1) and 12,680 (80.49%, CsY1) in KOG. The Pfam annotations included a set of candidate fungal pathogenicity-related genes, with CfC1 possessing more than CsY1 (Table 5). 3,531 and 3,616 pathogen-host interaction genes, 253 and 279 carbohydrate-active enzymes, 337 and 481 membrane transport proteins, 1,299 and 885 putative secreted proteins, 677 and 395 effectors, 90 and 59 secondary metabolite biosynthetic gene in CfC1 and CsY1, respectively (Table 5).

**Figure 5.**
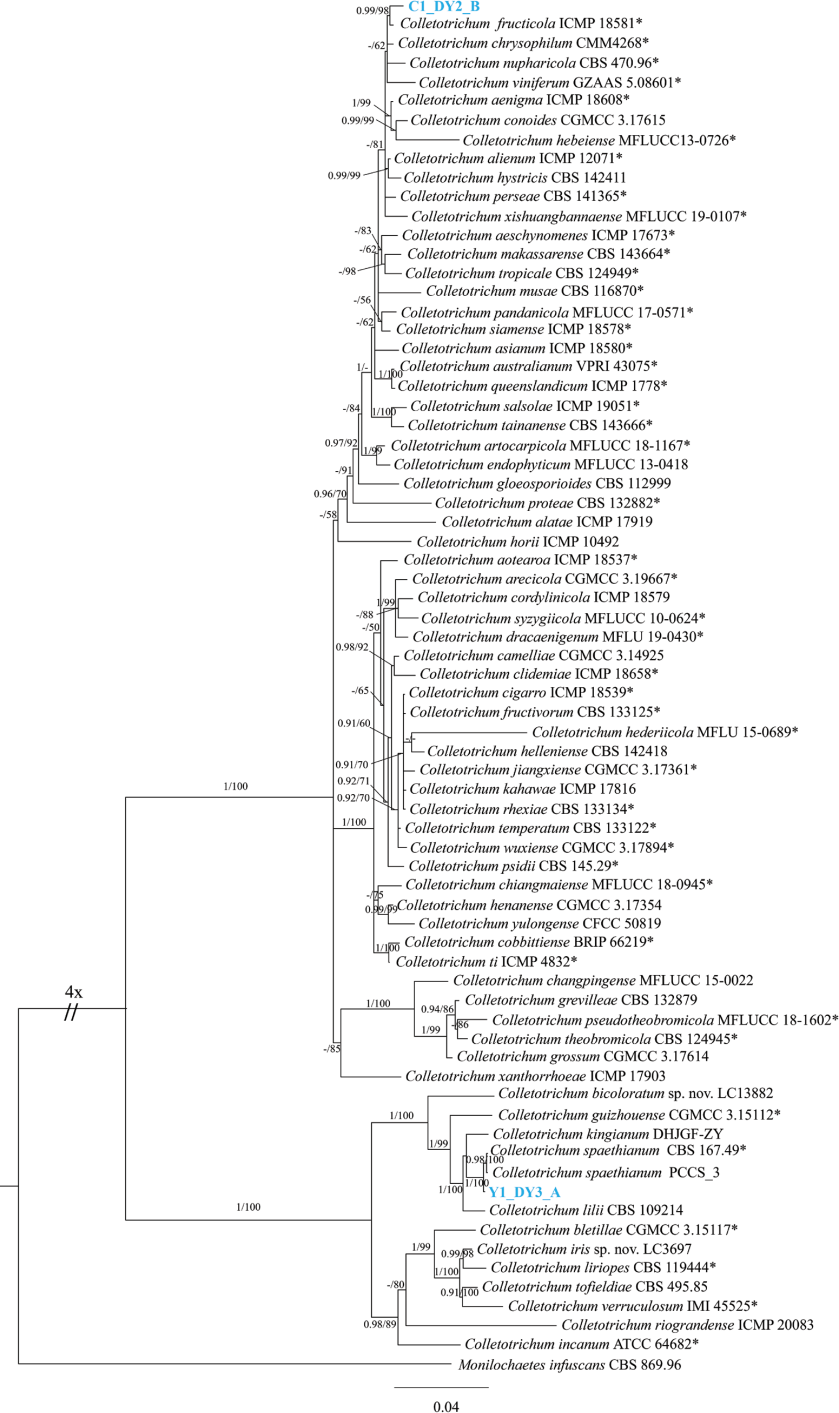
Bayesian inference phylogenetic tree of *Colletotrichum* sections *gloeosporioides* and *spaethianum* indicating the two sequenced isolates (in blue). *Monilochaetesinfuscans* (CBS 869.96) is used as outgroup. The tree was built using concatenated sequences of the ACT, CHS-1, GAPDH, HIS3, ITS and TUB2. Maximum likelihood bootstrap values ≥ 50%. Bayesian posterior probabilities ≥ 0.90 (PP /MLBS) are displayed on the phylogenetic tree.

**Figure 6.**
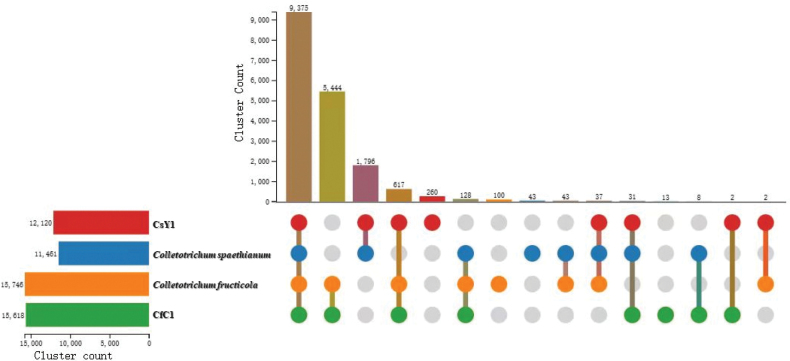
Protein level homologous gene cluster analysis of the genome of the two *Colletotrichum* species isolated from *Polygonatumcyrtonema*.

Phylogenetic analysis confirmed the identity of the two sequenced isolates (Fig. 5). The isolates of *C.spaethianum* Y1_DY3_A (CsY1) and *C.fructicola* C1_DY2_B (CfC1) were assigned to the respective species. We compared the two sequenced genomes with those from publicly available species in the same genus (the same genomes used for annotating protein-coding genes above). The 17,410 and 15,888 proteins in CfC1 and CsY1, respectively, formed 15,618 and 12,120 clusters. These included 17,899 non-redundant clusters which contained 7,783 single-copy clusters that were present in all *Colletotrichum* species and two species-specific clusters. Strains CfC1 and CsY1 shared more gene clusters with *C.fructicola* and *C.spaethianum*, respectively (Fig. 6), reflecting their closer evolutionary relationships. In conclusion, the high-quality genomes and annotated gene resources that we provided in this study will benefit future research on the infection and pathogenicity mechanisms of *C.fructicola* and *C.spaethianum*.

**Authors**: Xianzhi Zhou^*^, Yuan Wu, Yanping Hu, Hao Yu, Jianjin Zhou, Hongli Hu^*^

*Contact: xianzhizhou@126.com; huhongli7905@gmail.com

**Table 2.** Genome assembly features of *Colletotrichumspaethianum* strain Y1_DY3_A (CsY1) and *Colletotrichumfructicola* strain C1_DY2_B (CfC1).

Features	CsY1	CfC1
PacBio long reads (Gb)	8.51	18.81
Illumina short reads (Gb)	4.52	8.90
Estimated genome size (bp)	52,970,789	55,821,760
Assembly size (bp)	54,491,462	56,767,350
Contig number	107	13
Contig N_50_ (bp)	1,599,232	4,938,816
Contig N_90_ (bp)	319,619	4,303,157
Average contig length (bp)	509,266	7,595,691
Maximum contig length (bp)	4,068,025	7,120,155
GC content (%)	52.42	53.16

**Table 3.** Completeness and accuracy of the genome assemblies of the two *Colletotrichum* species isolated from *Polygonatumcyrtonema*.

Methods		CsY1		CfC1	
BUSCO	Database	Fungi*	Ascomycota*	*Fungi*	*Ascomycota*
	Completeness	98.55%	97.66%	97.63%	97.71%
	Single copy BUSCOs	744	1,660	733	1,653
	Duplicated BUSCOs	3	6	7	14
	Fragmented BUSCOs	1	2	1	9
	Missing BUSCOs	8	34	9	30
Mapping rates	PacBio long reads	99.19%		99.36%	
	Illumina short reads	99.36%		99.92%	
		(97.96% Properly paired)		(99.02% Properly paired)	
Merqury	Average base error	2.31 × 10^-4^		2.09 × 10^-5^	
		(QV = 36.36)		(QV = 46.81)	
	Genome completeness	97.92%		99.88%	

*: *Fungi* for fungi_odb10 (n = 758), *Ascomycota* for ascomycota_odb10 (n = 1,706).

**Table 4.** Repetitive sequence analysis of the genome of the two *Colletotrichum* species isolated from *Polygonatumcyrtonema*.

Repetitive sequence	Features	*C.spaethianum* Y1_DY3_A		*C.fructicola* C1_DY2_B	
		Length (bp)	Percentage%	Length (bp)	Percentage%
Interspersed repeats	SINEs	3,657	0.01	5,009	0.01
	LINEs	125,572	0.23	242,286	0.43
	LTR elements	2,405,105	4.41	241,689	0.43
	DNA transposons	148,291	0.27	491,988	0.87
	Unclassified	801,025	1.47	391,824	0.69
Tandem repeats	Small RNA	14,070	0.03	8,487	0.01
	Satellites	0	0.00	14,968	0.03
	Simple repeats	556,157	1.02	531,614	0.94
	Low complexity	59,823	0.11	58,036	0.10
	Total repeats	4,111,949	7.55	1,983,026	3.49

**Table 5.** Gene functional annotation of the genome of the two *Colletotrichum* species isolated from *Polygonatumcyrtonema*.

Annotation	*C.spaethianum* Y1_DY3_A		*C.fructicola* C1_DY2_B	
	Gene numbers	Percentage%	Gene numbers	Percentage%
Protein-coding genes	15,753	100.00	16,928	100.00
Pfam	11,456	72.72	11,547	68.21
GO	6,778	43.03	6,837	40.39
KEGG	6,428	40.80	5,121	30.25
KOG	12,680	80.49	12,378	73.12
CAZys	279	1.77	253	1.49
PHIs	3,616	22.95	3,531	20.86
Cytochrome P450 enzymes	158	1.00	279	1.65
Membrane transport proteins	481	3.05	337	1.99
Putative secreted proteins	885	5.62	1,299	7.67
Effectors	395	2.51	677	4.00
SMBGCs^#^	59	/	90	/

^#^: SMBGCs: secondary metabolite biosynthetic gene clusters.

## ﻿IMA GENOME-F 20D Draft genome sequence of *Neopestalotiopsismacadamiae* from macadamia in South Africa

### ﻿Introduction

*Pestalotiopsis*, a genus in the *Sordariomycetes* was reclassified in 2014 into 3 genera (*Pestalotiopsis*, *Neopestalotiopsis* and *Pseudopestalotiopsis*) based on morphology and phylogenetic analysis using the β-tubulin (β-tub), Internal Transcribed Spacer (ITS), and Translation Elongation Factor 1-α (tef1) gene regions (Maharachchikumbura et al. 2014). Since then, several *Neopestalotiopsis* species have been described and classified as plant pathogens and are associated with numerous diseases in various hosts, such as causing stem girdling and dieback on *Eucalyptus* (Diogo et al. 2021), grey leaf spot disease on *Mangiferaindica* (mango) (Gerardo-Lugo et al. 2020), grey blight leaf disease on *Camelliasinensis* (tea) (Chen et al. 2018; Wang et al. 2022) and leaf blight and crown rot on strawberry on *Fragaria×ananassa* (strawberry) (Rebollar-Alviter et al. 2020; Wu et al. 2020).

*Neopestalotiopsismacadamiae* represents an important pathogen of macadamia (*Macadamiaintegrifolia* and *M.tetraphylla*) grown in commercial plantations in tropical and subtropical frost-free regions in various countries around the world, including South Africa (Akinsanmi et al. 2017). This is because *N.macadamiae* causes dry flower disease (*Pestalotiopsis* blight) that results in yield loss by causing necrotic blight symptoms of the inflorescences and a dry appearance of the raceme (Akinsanmi et al. 2017). This pathogen causes yield loss and is regarded as one the most dominant and economically important threats to the macadamia industry (Akinsanmi et al. 2017).

Genome data are a valuable tool to understand the different aspects that can contribute towards developing control strategies for this pathogen. Currently, the pathogen is poorly understood and further research to understand various characteristics such as its plant host interactions, pathogenesis-related gene repertoire, and reproduction is required. *Neopestalotiopsisrosae* causes leaf blight and crown rot on strawberry and is the only publicly available genome for this important genus (Hsu et al. 2022; Han et al. 2024). The aim of this study was to generate the first draft genome assembly of a *N.macadamiae* strain, a confirmed pathogen of macadamia inflorescences through pathogenicity trials (Botha 2024), from South Africa. The pathogenic nature of several *Neopestalotiopsis* species makes it crucial to assemble high-quality reference genomes, which will provide a critical foundation for advancing research into the biology and pathogenic mechanisms of *Neopestalotiopsis* species.

### ﻿Sequenced strain

**South Africa**: Barberton: Mpumalanga, isolated from *Macadamiaintegrifolia* × *M.tetraphylla* inflorescences, 2022, B. Sonnekus (CMW 64591)

### ﻿Nucleotide sequence accession number

The Whole Genome Shotgun project of *Neopestalotiopsismacadamiae* CMW 64591 has been deposited at DBJ/EMBL/GenBank under the accession JBEXAB000000000. The version described in this paper is version JBEXAB000000000.

### ﻿Methods

The culture of isolate CMW 64591 was grown on MEA (malt extract agar; Biolab, South Africa) at 25 °C and 90% relative humidity with a 12-h photoperiod for 10 days. Genomic DNA was extracted from mycelia using the Quick-DNA Fungal/Bacterial Miniprep Kit (Zymo Research, California, USA) and submitted to Inqaba Biotechnical Industries (Pty) Ltd (Pretoria, South Africa) for whole genome sequencing using PacBio® Hifi Sequel IIe Sequencer. The quality of the sequenced long reads was assessed using FastQC v0.11.7 (Babraham Bioinformatics, Babraham Institute, Cambridge, UK). Canu v2.0 (Koren et al. 2017) was used for the *de novo* assembly and SeqKit v0.10.1 (Shen et al. 2016) was used to filter contigs smaller than 500 bp (Shen et al. 2016). Minimap2 (Li 2018) and SAMtools v. 1.18 (Li et al. 2009) were used to calculate the average base coverage by mapping the reads back to the genome. Genome statistics were determined using Quast v. 5.0.2 (Gurevich et al. 2013). The genome completeness was estimated using Benchmarking Universal Single-Copy Orthologs (BUSCO) in combination with the fungal data dataset (Simão et al. 2015). vRepeatModeler v2.0.1 (Flynn et al. 2020) was used to create a custom library of repeat families for the assembly to soft mask the assembly with RepeatMasker open-4.0.7 (Smit et al. 2013).

Genes were predicted in the masked assembly with Funannotate predict, which uses Augustus v3.5.0 (Stanke et al. 2006b), GeneMark-ES (Ter-Hovhannisyan et al. 2008), Glimmer v3.0.4 (Delcher et al. 2007), and SNAP (Korf 2004) as *ab initio* predictors. It also incorporates protein evidence in the form of BLAST alignments from the UniProt database (The UniProt Consortium 2023) and predicts tRNA genes with tRNAscan-SE v2.0.12 (Lowe and Eddy 1997). Functional annotations were assigned to the predicted genes by comparing them against multiple annotation databases. These included InterProScan v5.52–86.0 (Jones et al. 2014), EggNOG-mapper v2.1.11(Cantalapiedra et al. 2021), antiSMASH v6.1.1 (Blin et al. 2021) and Phobius v1.01 (Käll et al. 2007). These annotations were merged into a single file by running the Funannotate annotate pipeline, which additionally compared predicted proteins against the dbCAN v11.0 (Yin et al. 2012) database of carbohydrate-active enzymes (CAZymes) and the MEROPS v12.0 (Rawlings et al. 2014) protease database. To confirm the identity of the isolate, the β-tub, ITS and tef1 gene regions were extracted from the genome assembly. These sequences were included in a maximum likelihood (ML) phylogeny with closely related *Neopestalotiopsis* species obtained from GenBank (NCBI) using *Pestalotiopsisdiversiseta* MFLUCC12-0287 as an outgroup. The sequence datasets were aligned using an online version of MAFFT v. 7 (Katoh et al. 2019) and concatenated in Geneious Prime (Kearse et al. 2012). Phylogenetic analysis using ML was performed with RaxML v. 8 (Stamatakis 2014), based on the GTR substitution model with gamma-distribution rate variation and applying 1,000 bootstrap replicates. MITOS was used to identify and annotate the mitochondrial genome (Bernt et al. 2013).

### ﻿Results and discussion

The assembled genome size of *N.macadamiae* CMW 64591 was 50.05 Mb (for 0.5 kb+ scaffolds), consisting of 10 contigs and an approximated genome coverage of 22×. At 50.05 Mb, the *N.macadamiae* CMW 64591 genome is slightly smaller than the *N.rosae* ML1664 genome (53.78 Mb) (Hsu et al. 2022). However, the G/C content (50.22%) and the number of contigs (10) of *N.macadamiae* CMW 64591 is similar to the G/C content (49.88%) and the number of contigs (18) of *N.rosae* ML1664 genome.

The BUSCO analysis showed a completeness level of 99.4% with respect to the fungal dataset. Slightly more than the 98.4% BUSCO score observed for the *N.rosae* ML1664 genome (Hsu et al. 2022). In total, 454 ORFs occurred as single copies, 1 was duplicated, 0 were fragmented and 4 were missing from the *N.macadamiae* genome assembly. The Quast analysis indicated that the largest contig was 9,876,848 bp in length, a N50 of 7,068,772 bp, a N75 of 6,415,834 bp, a L50 score of 4 and L75 score 5 were observed. A total of 1.28% of the genome consisted of masked repeats. Contig 8 was identified as the mitochondrial genome of the *N.macadamiae* CMW 64591 assembly.

**Figure 7.**
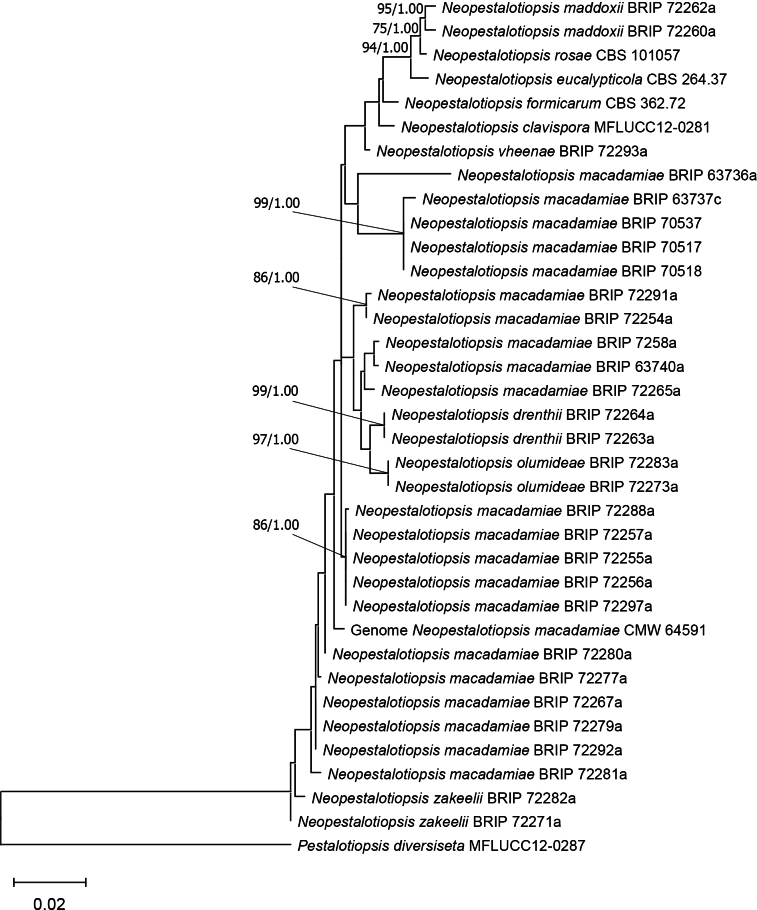
Maximum-likelihood (ML) tree topology of *Neopestalotiopsis* inferred on an alignment of the concatenated sequences of the β-tub, ITS and tef1 gene regions. *Pestalotiopsisdiversiseta* (MFLUCC 12-0287) was used as an outgroup taxon. The ML tree was based on the GTR substitution model with gamma-distribution rate variation. ML bootstrap support values (>75%) and Bayesian inference posterior probabilities (>90%) are displayed at the nodes.

A total of 14,221 genes was predicted of which 14,012 were functionally annotated. This included 829 CAZymes of which the AA7 and AA3 CAZyme families, which are involved in lignin degradation in plants, were most abundant. ML analysis of three barcoding gene regions grouped the genome isolate close to *N.macadamiae* isolates obtained from Australia (Fig. 7). The phylogenetic analysis also revealed species delineation issues and an increased availability of *Neopestalotiopsis* genomes could facilitate use of more informative gene regions to use for improved phylogenetic resolution. The draft genome sequence of *N.macadamiae* generated here will facilitate future research regarding the biology and pathogenicity of this fungus. Genome data will also facilitate improved understanding of secondary metabolite production, mating strategies, and the ecological interactions between *Neopestalotiopsis* species, and their plant hosts.

**Authors**: Byron Sonnekus^*^, Magriet A. van der Nest, Brenda D. Wingfield, Emma T. Steenkamp, Nicky Creux, Gerda Fourie

*Contact: Byron.Sonnekus@fabi.up.ac.za

## ﻿IMA GENOME-F 20E The first draft genome assembly of *Sphaerellopsisfilum*, a common mycoparasite of rust fungi

### ﻿Introduction

The genus *Sphaerellopsis* belongs to the family *Leptosphaeriaceae* in the order *Pleosporales*. *Eudarluca*, the typified name for the sexual morph of this genus is treated as a synonym (Trakunyingcharoen et al. 2014) and *Sphaerellopsis* is preferred over *Eudarluca* (Rossman et al. 2015). Eleven *Sphaerellopsis* species names are listed as legitimate in MycoBank; amongst these, seven species are supported with DNA barcode sequences in NCBI GenBank. The type species, *S.filum*, is a common mycoparasite of many rust fungi (Yuan et al. 1998, 1999; Kiss 2001; Driessen et al. 2004; Trakunyingcharoen et al. 2014; Sun et al. 2019). *Sphaerellopsisanomala*, *S.macroconidialis*, *S.melampsorinearum*, and *S.paraphysata* were also described as being associated with pustules of different rust species (Nag Raj 1993; Trakunyingcharoen et al. 2014; Gómez-Zapata et al. 2024). The conidiomata of *S.hakeae* were found inside rust pustules, but also occurring separately without any association to the rust (Crous et al. 2016). Two recently recognised species, *S.isthmospora* and *S.artemisiae*, were described as saprobes on dead plant materials (Phookamsak et al. 2019; Doilom et al. 2021).

*Sphaerellopsisfilum* is commonly found in uredinia and telia of diverse rust species in the field (Yuan et al. 1998, 1999; Driessen et al. 2004; Pei et al. 2010; Gordon and Pfender 2012) and some strains have been evaluated as potential biocontrol agents of some rusts (Pei and Yuan 2005; Durgadevi et al. 2024; Gómez-Zapata et al. 2024). Despite this, little is known about the ultrastructural aspects of this specific interfungal parasitic relationship (Plachecka 2005; Gómez-Zapata et al. 2024). Experiments revealed important differences in the mycohost specialisation and virulence of different *S.filum* strains (Yuan et al. 1999; Nischwitz et al. 2005; Pei et al. 2010). Phylogenetic analyses and molecular diversity studies indicated that *S.filum* strains are genetically diverse (Liesebach and Zaspel 2004; Nischwitz et al. 2005; Bayon et al. 2006).

Strikingly, *S.filum* is closely related to *Ampelomyces* spp. (Nischwitz et al. 2005; Trakunyingcharoen et al. 2014) that are widespread mycoparasites of powdery mildews (Kiss et al. 2011; Németh et al. 2021). This appears to be a rare example of the convergent evolution of mycoparasitism in close relatives. How this evolution has occurred and its genetic basis is unknown, but comparative genomics may provide insights. Genomic resources are already available for *Ampelomyces* (Haridas et al. 2020; Huth et al. 2021). However, as of 29 August 2024, there are no genome assemblies for any *Sphaerellopsis* spp. in GenBank. The aim of this study was, therefore, to provide the first draft genome assembly for *S.filum*. This resource will serve as the starting point to decipher the unique mode of action of *S.filum* against rust fungi, and the evolution of specific mycohost-mycoparasite interactions at the molecular level.

### ﻿Sequenced strain

*Sphaerellopsisfilum*: Portugal: isolated from *Pucciniahordei* on *Ornithogalumdivergens*, 1951, B. d’Oliveira (CBS 235.51 = ATCC 22604).

### ﻿Nucleotide sequenced accession number

The annotated genome sequence of *Sphaerellopsisfilum* CBS 235.51 has been deposited at GenBank under the accession JBFTXA000000000.

### ﻿Materials and methods

*Sphaerellopsisfilum* strain CBS 235.51 was obtained from the Westerdijk Fungal Biodiversity Institute (Utrecht, The Netherlands) and was maintained on potato dextrose agar (Amyl Media, Australia) at 18 °C in darkness. For DNA extraction, the strain was cultured in 20 ml Czapek-Dox broth (BD, Bacto Laboratories, Australia) in a Falcon tube and incubated on a shaker-incubator at 150 rpm for 2 weeks at 18 °C. Harvested mycelia were flash-frozen in liquid nitrogen and lyophilized for 48 hours. Eighty milligrams of lyophilized mycelia were ground with stainless steel beads (2.8 mm diameter, Sigma–Aldrich, Australia) in a Qiagen TissueLyser II (Qiagen, Australia) at 30 Hz/s for 30 s, flash-frozen in liquid nitrogen again and stored at -80 °C until DNA extraction. DNA was extracted using a Qiagen DNeasy Plant Mini Kit (Qiagen, Australia) following a modified protocol as described by Vaghefi et al. (2024). The internal transcribed spacers and the intervening 5.8S region were amplified using ITS1 and ITS4 primers (White et al. 1990), and submitted to Macrogen (Seoul, South Korea) for Sanger sequencing. Sequences were manually edited and consensus sequences were generated in Geneious Prime® 2024.0.5 (Biomatters Inc., New Zealand). Reference *Sphaerellopsis* sequences were downloaded from GenBank from the studies of Trakunyingcharoen et al. (2014), Crous et al. (2016), Phookamsak et al. (2019), Doilom et al. (2021), and Gómez-Zapata et al. (2024). A maximum likelihood phylogeny was constructed using RAxML v.8 (Stamatakis 2014) using a GTR GAMMA substitution model, and visualized in FigTree v.1.4.4 (http://tree.bio.ed.ac.uk/software/figtree/).

The same DNA sample used for the ITS amplification was used for Illumina library construction and Illumina NovaSeq sequencing (150 bp paired-end [PE]) at the Australian Genome Research Facility (AGRF, Melbourne, Australia).

Total RNA was extracted from two-week-old cultures grown in Czapek-Dox broth and incubated at room temperature on a shaker at 150 rpm. Fresh fungal mycelia were flash-frozen and ground in liquid nitrogen, then used for RNA extraction with an RNeasy Plant Mini Kit (Qiagen, Australia) following manufacturer’s instructions and including column-based DNase treatment. The extracted RNA was assessed via agarose gel electrophoresis and quantified using a Qubit v.4.0 fluorometer (ThermoFisher Scientific, Australia). The RNA transcriptome was sequenced by the AGRF (150 bp PE).

Raw sequence data were screened and filtered for contaminating sequences using Kraken v.2.1.2 (Wood et al. 2019) using the NCBI’s RefSeq archaea, viral, and UniVec_Core databases. Adapter removal from Illumina reads and quality trimming was conducted using BBduk from the BBmap suite v.39.03 (Bushnell 2014) (ktrim=r k=23 mink=11 hdist=1 tpe tbo qtrim=rl trimq=15). Genome assembly and error correction was conducted in SPAdes (Prjibelski et al. 2020) using read-correction and auto-*k*-mer selection options. The ITS sequence was extracted from the assembled genome by constructing a BLAST database in Geneious Prime and using the ITS sequence of the ex-neotype culture of *S.filum* (GenBank KP170657) as query against the genome. Assembly completeness was estimated using Benchmarking Universal Single-Copy Orthologs (BUSCO) v.5.5.0 (Manni et al. 2021a) against the *Dothideomycetes* database (dothideomycetes_odb10). For genome annotation, repeat content was determined using RepeatModeler v.2.0.1 (Flynn et al. 2020) and soft-masked using Repeatmasker using RepeatMasker v.4.1.0 (Smit et al. 1996–2010). Evidence-based genome annotation was conducted using BRAKER v.3 (Stanke et al. 2006a, 2008) using RNASeq data as evidence (Gabriel et al. 2021). Raw genome data were also used for genome size estimation in GenomeScope (Ranallo-Benavidez et al. 2020) Galaxy version 2.0.GC content distribution was determined using the software OcculterCut v.1.1 (Testa et al. 2016).

### ﻿Results and discussion

Using Illumina NovaSeq technology, 34,834,531 paired-end reads (10.5 Gb) were generated, 33,974,265 of which passed contamination and quality filtering (10 Gb). GenomeScope estimated a genome size of 28.9 Mb based on these DNA sequence data. *De novo* genome assembly of the *S.filum* strain included a total of 850 scaffolds larger than 250 bp and a draft genome size of 28,451,751 with 300× coverage (Table 6). Genome completeness based on Benchmarking Universal Single-Copy Orthologs in *Dothideomycetes* was estimated to be 95.5%, corresponding to 95.4% complete and single-copy BUSCOs, 0.1% complete and duplicated BUSCOs, 0.4% fragmented and 4.1% missing BUSCOs. Transcriptome sequencing generated 30,197,914 sequences (9.1 Gb), 99% of which passed quality control. A total of 9,626 genes including 11,671 coding sequences (CDS) were predicted in the *S.filum* CBS 235.51 assembly.

**Figure 8.**
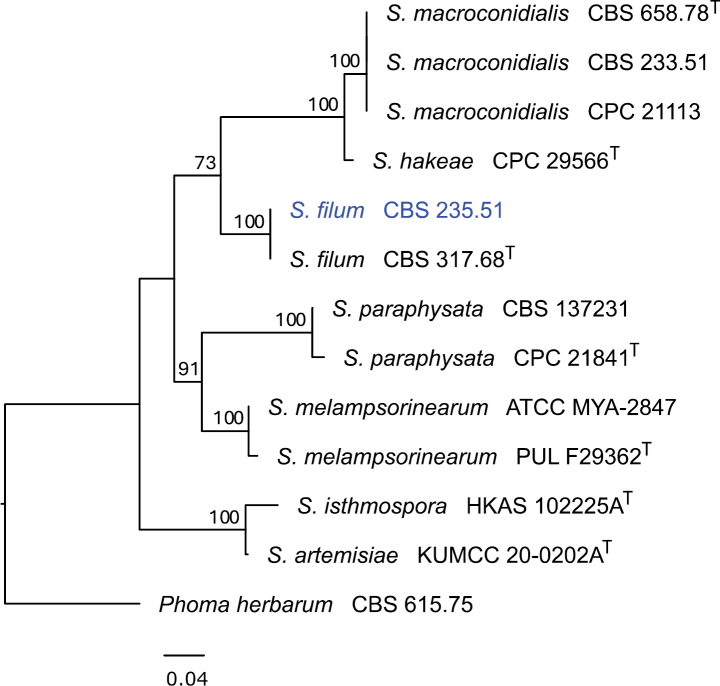
Phylogenetic tree of *Sphaerellopsis* species with available sequence data, based on maximum likelihood analysis of the ITS region. Analyses were performed in Geneious Prime v.2024.0.5 using RAxML v. 8.2.11 (Stamatakis 2014) based on the GTR substitution model with gamma-distribution rate variation. Bootstrap support values (from 1,000 reiterations) greater than 70% are given at the nodes. The tree was rooted to *Phomaherbarium* CBS 615.75. The isolate sequenced in this study is shown in blue and ex-type strains are indicated with T.

The ITS sequence of CBS 235.51 produced using Sanger sequencing (GenBank accession PP972725) was identical to the ITS sequence of CBS 317.68 (GenBank KP170657), the ex-neotype culture of *S.filum*. The ITS sequence extracted from the Illumina genome sequencing of CBS 235.51 was identical to this sequence. The phylogenetic placement of strain CBS 235.51 based on the ITS sequence is provided in Fig. 8. This confirmed the identity of the genome-sequenced isolate as *S.filum*.

This is the first genome assembly of a *Sphaerellopsis* strain, which revealed a smaller genome size compared to *Ampelomyces* spp., another group of mycoparasitic fungi within *Pleosporales* (Table 6). Analysis of the assembled genome in Occultercut revealed its bipartite structure, similar to *Ampelomyces* species (Huth et al. 2021). The percentage of AT-rich regions in the assembled genome of CBS 235.51 (10%) is, however, lower compared to *Ampelomyces* species (Huth et al. 2021; Table 6). These AT-rich regions, which are also prevalent in many plant pathogeneic fungi, are believed to be the result of repeat-induced point mutations, which is a defence mechanism against propagation of transposable elements in fungi (Testa et al. 2021). This could be the underlying reason for the fragmentation of the assembled genome and indicates a need for long-read sequence data to further sequence and assembled these repetitive regions. In the meantime, the availability of the first genomic resources for this important and widespread mycoparasitic species will facilitate research focussed on their evolution, ecology, and mycoparasitic activity.

**Authors**: Niloofar Vaghefi*, Jason Risteski, Alexander Idnurm, Levente Kiss

*Contact: vaghefin@unimelb.edu.au

**Table 6.** Genome statistics and comparison with *Ampelomyces* species.

Strain	Host species	Sequencing technology	Assembly size (Mb)	Cov^a^	No. of scaffolds >500 bp	N50 (Mb)	GC content (%)	Genome Completeness (%)^b^	Percentage of AT-rich regions^c^	NCBI Accession Number	Reference
CBS 235.51	*Pucciniahordei*	Illumina paired-end NovaSeq	28.5	300×	330	375,353	49.7	95.5	10.1	JBFTXA000000000	This study
BRIP 72107	*Golovinomycesbolayi*	Illumina paired-end MiSeq and Oxford Nanopore MinION	40.4	400×	24	2,994,887	45.5	96.6	33.9	JAGTXZ000000000	Huth et al. (2021)
HMLAC 05119	Undetermined powdery mildew	Illumina paired-end and mate-pair	36.8	103×	464	258,565	46.5	96.3	26.6	VOSX00000000.1	Haridas et al. (2020)

^a^ Genome coverage. ^b^ Assembly completeness for all the genomes was determined based on Benchmarking Universal Single-Copy Orthologs (BUSCO) v.5.5.0 (Manni et al. 2021a) against the dothideomycetes_odb10 database. ^c^ Denotes the percentage of repeat-rich regions with reduced GC content, estimated by OcculterCut (Testa et al. 2021).

## ﻿IMA GENOME-F 20F Genome sequence of *Pyrenopezizabrassicae*, causal organism of light leaf spot disease on *Brassica* species

### ﻿Introduction

*Pyrenopezizabrassicae* Sutton and Rawlinson is an air-borne, extracellular fungal pathogen of *Brassica* species. It has been taxonomically accommodated within the family *Ploettnerulaceae* of the order *Helotiales* within the phylum *Ascomycota*. This pathogen was also known by its anamorph, *Cylindrosporiumconcentricum*, for many years; this was initially described by Greville (1823) considering the concentric ring-like pattern of the asexual sporulation. Ascomata (sexual sporulating structures) of *P.brassicae* were first observed on culture media by Thomson (1936) and Cabral (1940), and later described as immature apothecia by Hickman et al. (1955). The occurrence of *P.brassicae* apothecia under natural conditions was first reported by Staunton and Kavanagh in 1966, based on their observations in diseased vegetable brassica crops in Ireland. The precise identification of *P.brassicae* was made by Rawlinson et al. (1978), who described it as the teleomorph of *Cylindrosporiumconcentricum* (Rawlinson et al. 1978; Cheah et al. 1980).

The pathogen thrives in areas with cool, wet climates and shows a wide geographic distribution, occurring in many regions of the world including the UK, continental Europe, Japan, New Zealand and North America (Boys et al. 2007; Karolewski et al. 2012; Karandeni Dewage et al. 2018; Carmody et al. 2020). In the UK, light leaf spot disease caused by *P.brassicae* is considered as one of the most economically damaging diseases of winter oilseed rape (*Brassicanapus*) with yield losses potentially up to 30–50% (Rawlinson et al. 1978; AHDB 2021). The steady increase in the severity of light leaf spot epidemics since the early 2000s in England, with it replacing phoma stem canker as the main disease of winter oilseed rape there, has increased the economic importance of *P.brassicae*. There have been severe light leaf spot epidemics affecting oilseed rape crops in other parts of northern continental Europe and Poland and vegetable brassica crops in New Zealand (Boys et al. 2007; Karandeni Dewage et al. 2018; Bucur et al. 2022).

Thorough understanding of the underpinning molecular mechanisms and the genetic basis of host-pathogen interactions is a key to successful management of light leaf spot. However, there is little information available on the genes involved in *P.brassicae* pathogenicity, and no published whole genome sequence of *P.brassicae* is available at present. Also, a genome sequence can provide new tools to study the population structure of this pathogen, since there may be variations in pathogenicity between isolates from different geographic regions (Karandeni Dewage et al. 2018). According to Carmody et al. (2020), *P.brassicae* from North America is considered as a different lineage from those present in other geographic regions. The genome sequence of this pathogen is an invaluable resource to investigate these taxonomic/evolutionary identities. There will also be new possibilities for comparative genomic studies since *P.brassicae* is known to have a close evolutionary relationship with eyespot pathogens on cereals (*Oculimaculayallundae* and *O.acuformis*) and leaf blotch (also called leaf scald) pathogen *Rhynchosporiumcommune* (formerly known as *R.secalis*), on barley (Goodwin 2002).

Considering its economic and evolutionary importance, we present the *de novo* whole genome sequence of *P.brassicae*. This will enable new studies of the biology and genetics of this pathogen, providing knowledge to improve current light leaf spot management strategies and address evolutionary questions.

### ﻿Sequenced isolate

*P.brassicae* isolate 15WOSR64-SS1 was obtained from diseased leaves of oilseed rape (*Brassicanapus*) cultivar Bristol from Hereford, UK in 2015 (Karandeni Dewage et al. 2021). The mating type of this isolate was confirmed to be *MAT1-1* by mating-type PCR (Foster et al. 2002).

### ﻿Nucleotide sequence and accession number

The whole genome sequence of *P.brassicae* (isolate 15WOSR64-SS1) has been deposited at the European Nucleotide Archive (ENA) under the accession number GCA_958299125.

### ﻿Materials and methods

A single conidial isolate (15WOSR64-SS1) of *P.brassicae* (Karandeni Dewage et al. 2021) was grown in potato dextrose broth (Oxoid Ltd., England) at 18 °C in a shaking incubator at 120 rpm. Mycelia were harvested from three-week-old cultures and freeze-dried. Genomic DNA was extracted from freeze-dried mycelia using the CTAB DNA extraction method for high molecular weight genomic DNA (Inglis et al. 2018). Purity of the extracted DNA was tested with a Nanodrop ND-1000 spectrophotometer (Labtech International, UK) and the DNA concentration was measured using a Qubit™ 3.0 fluorometer (Fisher Scientific, UK). Quality of the extracted DNA was assessed using a genomic DNA ScreenTape assay with Agilent 4150 Tape­Station (Agilent Technologies, UK) to estimate the integrity of the DNA, before sending the samples to GENEWIZ Europe (Leipzig, Germany) for sequencing.

The *P.brassicae* genome was sequenced using both the Illumina NovaSeq sequencing platform and a single PacBio Sequel SMRT cell at GENEWIZ Europe (Leipzig, Germany). The single paired-end Illumina NovaSeq library was prepared with an insert size of c. 350–450 bp and 150 bp read length. The PacBio DNA library was prepared with an insert size of 20 kbp with Blue Pippin size selection as per the manufacturer’s protocol. The prepared PacBio library was sequenced on the PacBio Sequel platform with v2.0 chemistry. The hybrid *de novo* assembly was done using the MaSuRCA 3.4.0 genome assembly and analysis toolkit (Zimin et al. 2017), utilizing both Illumina and PacBio-sequenced data. Illumina reads were merged and contigs of length greater than 250 bp were used for analysis. The normalization and read correction steps made use of MaSuRCA’s built-in functionalities. QUAST (Gurevich et al. 2013) was used to generate statistics for the *de novo* assembled genome. MaSuRCA’s implementation of Jellyfish was utilized for clustering assemblies based on *k*-mer estimations of the expected genome size. Finally, Flye (Kolmogorov et al. 2019) was used to construct the polished contigs. Completeness of the assembled genome was assessed using the Benchmarking Universal Single-Copy Orthologs tool (BUSCO) with BUSCO v4.1.3 (Simão et al. 2015) against ascomycota-odb10 and leotiomycetes_odb10 datasets.

To obtain *P.brassicae* RNA sequencing (RNA-seq) data, fungal mycelia harvested from two liquid cultures of the isolate 15WOSR64-SS1 (grown as described above) were snap frozen with liquid nitrogen and sent to GENEWIZ Europe (Leipzig, Germany), where total RNA was extracted using the RNeasy Plant Mini Kit according to the manufacturer’s protocol. Purity of the extracted RNA was tested with a Nanodrop 2000 spectrophotometer and the concentration was measured using a Qubit™ fluorometer. Further quality assessment was done using an RNA ScreenTape assay, followed by cDNA synthesis, library preparation and sequencing using the Illumina HiSeq sequencing platform at GENEWIZ Europe. Raw Illumina reads were trimmed using skewer v0.2.2 (Jiang et al. 2014) and *de novo* transcriptome assembly was done using Trinity v2.11.0 (Grabherr et al. 2011). Transdecoder was used to identify protein coding regions.

Structural annotation of the genome was done at DEEP Seq (Queen’s Medical Centre, University of Nottingham, Nottingham) using the MAKER annotation pipeline v2.31.11 (Holt and Yandell 2011) incorporating assembled transcripts and predicted peptides. SNAP (Korf 2004) and AUGUSTUS v3.4.0 (Stanke and Waack 2003) gene predictions were applied in the MAKER pipeline v2.31.11. Gene models from the closely related species, *R.commune*, genome (NCBI_Assembly:GCA_900074885.1) were also included into training of gene predictors. Repeat sequences were identified via MAKER using RepeatMasker 4.1.5 (Smit et al. 2013–2015) and tRNA (transfer RNA) and snoRNA (small nucleolar RNA) genes were predicted using tRNAscan-SE v2.0.9 (Chan et al. 2021) and snoscan 0.9.1 (Lowe and Eddy 1999), respectively. Functional annotation of the genes was done using Interproscan v5.0 (Jones et al. 2014) and BlastP (Altschul et al. 1990) against UniProt/SwissProt database.

To confirm the identity of the newly sequenced genome, phylogenetic analysis was done using nucleotide sequences of the internal transcribed spacers (ITS), translation elongation factor 1 (TEF1) and the beta tubulin genes. Publicly available nucleotide sequences of related fungal species and other *P.brassicae* isolates were obtained from the National Center for Biotechnology Information (NCBI) database and compared with those extracted from *P.brassicae* isolate 15WOSR-64SS1. Multiple alignments of ITS, TEF1 and beta tubulin nucleotide sequences were produced using MUSCLE (Edgar 2004). For the phylogenetic analysis of ITS sequences, all positions with less than 20% site coverage were eliminated and the Kimura 2-parameter (K2+G) model was used. For the beta-tubulin and TEF1 sequences, the Tamura-Nei (TN93+G) model was used after eliminating all positions with less than 60% and 30% site coverage, respectively. A maximum likelihood phylogenetic tree was produced by the program MEGA 11 (Tamura et al. 2021).

### ﻿Results and discussion

**Figure 9.**
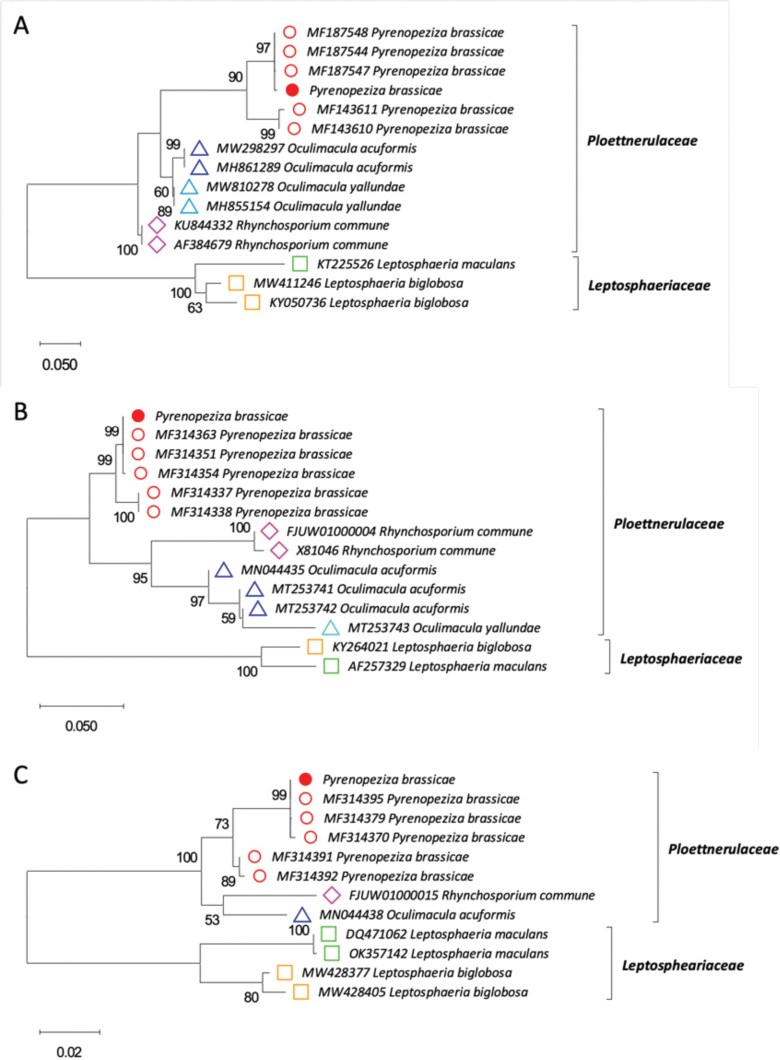
Phylogenetic analysis of the newly sequenced *Pyrenopezizabrassicae* and related fungi based on the nucleotide sequences of their (**A**) internal transcribed spacers (ITS), (**B**) beta-tubulin and (**C**) translation elongation factor 1 (TEF1). Phylogenetic analysis was done using nucleotide sequences of five related fungal species: *Rhynchosporiumcommune* (isolate IDs - 765.03.01, R157, UK7), *Oculimaculaacuformis* (isolate IDs - 22-418, 22-443, 22-498, 22-499, CBS 495.80), *Oculimaculayallundae* (isolate IDs - 22-495, CBS 128.31, CBS 110665), *Leptosphaeriamaculans* (isolate IDs - AFTOL-ID 277, Pk4), *L.biglobosa* (15PL-42, Azad4, Khal12) and of *P.brassicae* (isolate IDs – 233716, Cyc001, Cyc007, PC18, PC38). A multiple alignment of nucleotide sequences was produced using MUSCLE (Edgar 2004). For the phylogenetic analysis of ITS sequences, all positions with less than 20% site coverage were eliminated and the Kimura 2-parameter (K2+G) model was used. For the beta-tubulin and TEF1 sequences, the Tamura-Nei (TN93+G) model was used after eliminating all positions with less than 60% and 30% site coverage, respectively. A maximum likelihood phylogenetic tree was produced by the program MEGA 11 (Tamura et al. 2021). Bootstrap percentages (1,000 replicates) are shown. Different genera are indicated by different shapes and different species by different colours. Tips labelled with open shapes indicate amino acid sequences available in public databases. The red spot indicates the newly sequenced *P.brassicae*.

The genome sequence of *P.brassicae* was assembled into 45 scaffolds larger than 1,000 bp with a total genome size estimated to be 73.24 Mb. Of the 45 scaffolds, 38 were larger than 50,000 bp and the PacBio reads provided an estimated genome coverage of 95×. The genome had an average GC content of 42.85%, N50 value of 3,083,431 bp and L50 value of 11. BUSCO analysis against the ascomycota_odb10 dataset indicated that the assembled genome was 98.4% complete, identifying 1,684 out of 1,706 BUSCOs as present in the genome assembly (1,679 complete and single-copy, 2 complete and duplicated, 3 fragmented and 22 missing BUSCOs). BUSCO analysis against the leotiomycetes_odb10 dataset identified 3,181 out of 3,234 BUSCOs as present in the genome assembly (3,168 complete and single-copy, 1 complete and duplicated, 13 fragmented and 53 missing BUSCOs) with the genome completeness score of 97.9%. Table 7 summarises the key statistics of the *de novo* assembled genome. A total of 25,717 genes was predicted using the MAKER annotation pipeline, of which 23,620 were protein-coding genes. There were 143 tRNA genes and 1,954 snoRNA genes predicted. The phylogenetic trees (Fig. 9) based on the internal transcribed spacers (ITS), translation elongation factor 1 (TEF1) and beta tubulin nucleotide sequences confirmed the taxonomic identity of the sequenced isolate as *P.brassicae*.

This genome assembly is the first genome sequence of *P.brassicae* available. Considering the increased importance of this pathogen in recent years in the UK and North America, this genome resource will help to gain better understanding of *P.brassicae* and guide the development of more effective and efficient control strategies for light leaf spot. Control of crop diseases plays a key role in maintaining agricultural production for food security. Recently, genomic resources have accelerated the research on host-pathogen interactions, making considerable improvements to crop disease control. Regarding the hosts, the genome sequences of five *Brassica* species have been made available, including *B.rapa* (Wang et al. 2011), *B.oleracea* (Liu et al. 2014), *B.napus* (Chalhoub et al. 2014), *B.juncea* and *B.nigra* (Yang et al. 2016). In contrast to the host genomic information, there is very little information about the *P.brassicae* genome. *De novo* sequencing of the *P.brassicae* genome will therefore fill this resource gap and help to understand molecular and genetic basis of its interactions with host *Brassica* species. Additionally, considering the close evolutionary relationship of *P.brassicae* to major pathogens of cereal crops, such as *R.commune* and *Oculimacula* spp., this genome will be of a wide scientific interest, as it can pave the way to new comparative genomic studies.

**Authors**: Chinthani S. Karandeni Dewage*, Loly I. Kotta-Loizou, Henrik U. Stotz, Bruce D. L. Fitt, Yongju Huang

*Contact: c.s.karandeni-dewage@herts.ac.uk

**Table 7.** *Pyrenopezizabrassicae* 15WOSR-64SS1 genome assembly statistics.

Metric	Value
Number of contigs	45
Total length (bp)	73,238,495
Largest contig (bp)	4,725,742
GC content (%)	42.81
Number of contigs (>= 1,000 bp)	45
Number of contigs (>= 5,000 bp)	43
Number of contigs (>= 10,000 bp)	42
Number of contigs (>= 25,000 bp)	39
Number of contigs (>= 50,000 bp)	38
N50 (bp)	3,083,431
N75 (bp)	1,656,915
L50	11
L75	18
Number of N’s per 100 kbp	0.14
Predicted gene models	25,717
BUSCO completeness (%)	
ascomycota_odb10	98.4
leotiomycetes_odb10	97.8

## ﻿Additional information

### Author ORCIDs

Davide D’Angelo https://orcid.org/0009-0007-6542-6298

Tiphany Nkomo https://orcid.org/0000-0003-3355-5080

Xianzhi Zhou https://orcid.org/0000-0002-3734-075X

Niloofar Vaghefi https://orcid.org/0000-0003-0430-4856

Byron Sonnekus https://orcid.org/0000-0002-7228-4810

Tanay Bose https://orcid.org/0000-0002-2069-042X

Nicky Creux https://orcid.org/0000-0002-4179-6995

Nunzio D’Agostino https://orcid.org/0000-0001-9840-3817

Gerda Fourie https://orcid.org/0000-0003-2650-5448

Almuth Hammerbacher https://orcid.org/0000-0002-0262-2634

Alexander Idnurm https://orcid.org/0000-0001-5995-7040

Levente Kiss https://orcid.org/0000-0002-4785-4308

Emma T. Steenkamp https://orcid.org/0000-0003-0217-8219

Magriet A. van der Nest https://orcid.org/0000-0001-6914-8343

Chanthani S. Karandeni Dewage https://orcid.org/0000-0001-6621-8203

Brenda D. Wingfield https://orcid.org/0000-0002-6189-1519

### Data availability

All of the data that support the findings of this study are available in the main text or Supplementary Information. This includes cited data base accessions.

## Supplementary materials

Supplementary material 1 List of protein-coding genes identified in *A.rolfsii* strain CCF1 and their annotations Data type xlsx https://binary.pensoft.net/file/1253334This dataset is made available under the Open Database License (http://opendatacommons.org/licenses/odbl/1.0/). The Open Database License (ODbL) is a license agreement intended to allow users to freely share, modify, and use this Dataset while maintaining this same freedom for others, provided that the original source and author(s) are credited.Davide D’Angelo, Roberto Sorrentino, Ernesto Lahoz, Domenico Cerrato, Maurizio Viscardi, Loredana Cozzolino, Giovanna Fusco, Nunzio D’Agostino
